# Effects of Heparan sulfate acetyl-CoA: Alpha-glucosaminide N-acetyltransferase (HGSNAT) inactivation on the structure and function of epithelial and immune cells of the testis and epididymis and sperm parameters in adult mice

**DOI:** 10.1371/journal.pone.0292157

**Published:** 2023-09-27

**Authors:** Lorena Carvelli, Louis Hermo, Cristian O’Flaherty, Richard Oko, Alexey V. Pshezhetsky, Carlos R. Morales

**Affiliations:** 1 IHEM-CONICET, Universidad Nacional de Cuyo, Mendoza, Argentina; 2 Facultad de Ciencias Exactas y Naturales, Universidad Nacional de Cuyo, Mendoza, Argentina; 3 Department of Anatomy and Cell Biology, McGill University, Montreal, Quebec, Canada; 4 Department of Surgery (Urology Division), McGill University, Montréal, Quebec, Canada; 5 Department of Pharmacology and Therapeutics, McGill University, Montréal, Canada; 6 Department of Biomedical and Molecular Sciences, Queen’s University, Kingston, Canada; 7 Sainte-Justine University Hospital Research Center, University of Montreal, Montreal, Quebec, Canada; University of Hyderabad, INDIA

## Abstract

Heparan sulfate (HS), an abundant component of the apical cell surface and basement membrane, belongs to the glycosaminoglycan family of carbohydrates covalently linked to proteins called heparan sulfate proteoglycans. After endocytosis, HS is degraded in the lysosome by several enzymes, including heparan-alpha-glucosaminide N-acetyltransferase (HGSNAT), and in its absence causes Mucopolysaccharidosis III type C (Sanfilippo type C). Since endocytosis occurs in epithelial cells of the testis and epididymis, we examined the morphological effects of *Hgsnat* inactivation in these organs. In the testis, *Hgsnat* knockout (*Hgsnat-Geo*) mice revealed statistically significant decrease in tubule and epithelial profile area of seminiferous tubules. Electron microscopy (EM) analysis revealed cross-sectional tubule profiles with normal and moderately to severely altered appearances. Abnormalities in Sertoli cells and blood-testis barrier and the absence of germ cells in some tubules were noted along with altered morphology of sperm, sperm motility parameters and a reduction in fertilization rates *in vitro*. Along with quantitatively increased epithelial and tubular profile areas in the epididymis, EM demonstrated significant accumulations of electrolucent lysosomes in the caput-cauda regions that were reactive for cathepsin D and prosaposin antibodies. Lysosomes with similar storage materials were also found in basal, clear and myoid cells. In the mid/basal region of the epithelium of caput-cauda regions of KO mice, large vacuolated cells, unreactive for cytokeratin 5, a basal cell marker, were identified morphologically as epididymal mononuclear phagocytes (eMPs). The cytoplasm of the eMPs was occupied by a gigantic lysosome suggesting an active role of these cells in removing debris from the epithelium. Some eMPs were found in proximity to T-lymphocytes, a feature of dendritic cells. Taken together, our results reveal that upon *Hgsnat* inactivation, morphological alterations occur to the testis affecting sperm morphology and motility parameters and abnormal lysosomes in epididymal epithelial cells, indicative of a lysosomal storage disease.

## Introduction

A dense layer of glycoproteins and glycolipids, referred to as the glycocalyx, coats the surface of cells and is composed of various polysaccharides covalently linked to lipids or proteins [1–3]. Heparan sulfate (HS) belongs to the glycosaminoglycan (GAG) family of carbohydrates that are covalently linked to core proteins, collectively known as heparan sulfate proteoglycans (HSPGs). The GAG molecules are long, unbranched polysaccharides containing repeating disaccharide units. The most common repeating disaccharide unit of HS comprises a glucuronic acid or L-iduronic acid linked to an N-acetyl glucosamine via a β1,4 glycosidic bond [4–6]. These groups are sulfated at different N-, 2-O-, 6-O-, and 3-O-positions leading to structural diversity.

The ubiquitous presence of HSPGs in different tissues and organs of the body indicates the multitude of critical biological roles they play within an organism, which in addition to cell surface coats, include an integral basement membrane constituent and scaffolding component of the extracellular matrix [7–9]. HSPGs provide a propensity for interaction with a wide array of ligands and cognate receptors [3, 10–15] and also act as crucial components of protein binding within adjacent intracellular regions [16–21].

Lysosomal storage disorders (LSDs) comprise a heterogeneous group of rare inherited metabolic diseases characterized by accumulation of macromolecules inside lysosomes. LSDs are caused by deficiencies in lysosomal enzymes, leading to altered recycling of macromolecules, and impairment of the endolysosomal system [22–26]. Mucopolysaccharidoses (MPS) are a group of LSDs accounting for approximately 30% of all cases and arise from mutations in genes involved in glycosaminoglycans (GAGs) degradation [27, 28]. Under normal conditions, heparan sulfate glycosaminoglycan degradation proceeds in a stepwise fashion with the help of several enzymes, including three exoglycosidases, four sulfatases and an acetyltransferase [29]. Based on which enzyme is deficient, four different subtypes of MPS type III (MPS IIIA-D) have been recognized [30–33]. Mucopolysaccharidosis Type III C (MIM #252930) is caused by the deficiency of the lysosomal membrane enzyme, heparan sulfate acetyl-CoA: alpha-glucosaminide N-acetyltransferase (HGSNAT, EC 2.3.1.78) [30, 34–36].

HGSNAT is an integral lysosomal membrane protein that is not a hydrolase [37]. HGSNAT transfers an acetyl group from cytoplasmic-derived acetyl-CoA to terminal N-glucosamine residues of heparan sulfate within lysosomes before their subsequent hydrolysis by α-N-acetylglucosaminidase [34, 35, 38]. With a non-functional HGSNAT, heparan sulfate cannot be degraded and accumulates in the lysosomes of affected cells [30, 36, 39, 40].

The role of HS in the central nervous system has been well documented, with alterations in its breakdown by appropriate enzymes resulting in lysosomal storage diseases causing several neurodegenerative mucopolysaccharidoses [33, 34, 41–43]. However, analysis of the importance of the effect of HS storage on the structural integrity of the epithelial cells of the testis and epididymis are lacking, even though it has been reported to be abundant in these tissues [44–48]. This contrasts the case for other lysosomal storage diseases (LSDs) such as Niemann-Pick [49, 50], Tay-Sacks and Sandhoff diseases [51–53] for which a dramatic impact of storage materials on the morphology and functional parameters of the testis and/or epididymis is well documented.

Morphologically, the seminiferous epithelium of the testis contains germ cells that undergo mitotic (spermatogonia) and meiotic (spermatocytes) divisions followed by a non-dividing phase whereby the resulting germ cells, referred to as spermatids, undergo metamorphosis to yield sperm [54–57]. The somatic non-proliferating Sertoli cells form specific compartments within the epithelium via a blood-testis barrier, which monitors the production and development of germ cells [58–61]. Once produced, sperm enters the epididymis, an organ hovering alongside the testis and formed of a highly coiled tube consisting of a pseudostratified epithelium bordering a central lumen. It is subdivided arbitrarily into 4 regions, i.e. initial segment, caput, corpus and cauda, with each revealing structural and regional differences of its cell types and arbitrarily defined subdivisions [62–64]. Epithelial cells correspond to principal, narrow, apical, clear and basal cells [65–67], along with immune cells [66, 68, 69] initially described as wandering halo cells [70]. In recent years a well-defined population of epididymal mononuclear phagocytes (eMPS) consisting of monocytes/macrophages (M/M) and dendritic cells has also been documented at the base of the epithelium with essential immunological functions [71–74].

The importance of the length, region-specific epithelial cells modifications and duration of sperm presence within each region is critical for epididymal functions related to sperm maturation. In addition to its ability to render motility and fertility to the sperm, the duct also transports, concentrates, stores, and protects sperm [65–67, 75–77].

In the present study, we analyzed the effect of the *Hgsnat* gene inactivation in the testis and epididymis of mice. Abnormalities in the morphology of Sertoli and germ cells in some tubular cross-sections were noted in the knockout animals, along with altered morphology and motility parameters of sperm and reduced fertilized oocytes *in vitro*. Concomitantly, the epididymis revealed significant accumulations of lysosomal storage vacuoles in epithelial principal cells in a region-specific manner, along with lysosomal alterations of basal and clear cells and lysosomal engorgement of immune epididymal mononuclear phagocytes (eMPs) at the base of the epithelium.

## Materials and methods

### Animals

C57Bl6 mice with a targeted disruption of the *Hgsnat* gene (*Hgsnat*-/-) were generated using a gene trap approach [30]. The principal element of this trap vector was a gene trapping cassette consisting of a promoterless reporter gene and a selectable genetic marker, flanked by an upstream 3’ splice site and a downstream polyadenylation transcriptional termination sequence. The trapping cassette was inserted into the intron 7 of the *Hgsnat* gene. Since transcription is terminated prematurely at the inserted polyadenylation site, the processed fusion transcript encodes a truncated, non-functional version of the protein. The *Hgsnat-/-* male mice were compared with the appropriate age wild-type controls (C57Bl6, *Hgsnat+/+*). Mice were maintained under constant temperature and humidity, on a 12-hour light/ 12-hour dark cycle, with food and water ad libitum. Mice were sacrificed by a carbon dioxide euthanasia chamber, except when tissues were prepared for light and electron microscopy, which information is detailed below. All animal experiments were approved by the CHU Sainte-Justine Research Ethics Committee (Montreal QC) and performed in compliance with the Comité Institutionnel des Bonnes Pratiques Animales en Recherche (CIBPAR; approval number 2022–3453), in accordance with the Canadian Council on Animal Care (CCAC) guidelines.

### Tissue preparation: Light and electron microscopy

Within each age group, 3 *Hgsnat+/+* and 3 *Hgsnat*−/− mice were examined at 7, 11 and 14 month-old. Before experimentation, mice were anesthetized with an intraperitoneal injection of sodium pentobarbital (Somnitol, MTC Pharmaceuticals, Hamilton, ON).

### Light Microscopy (LM)

Blood vessels and the efferent ducts (EDs) at their junction with the testis (rete testis) from the right side of each animal were clamped with a hemostat and cut, allowing the removal of the efferent ducts and epididymis from the testis. The efferent ducts were dissected free from the enveloping fat. At the same time, the epididymis was cut in half along its long axis to reveal its 4 major regions, i.e., initial segment (IS), caput, corpus and cauda [78]. The EDs and epididymis were immersed in Bouin’s fixative (BD Biosciences, Mississauga, ON; Beckstead) for 24 hours. The right testis was punctured with a syringe, and 10 ml of Bouin’s fixative was administered into its interior under the tunica albuginea. After a 1 hour duration, the testis was cut in half and immersed in a solution of the fixative for 24 hours. The following day, all tissues were dehydrated through a graded ethanol series and eventually embedded in paraffin. Sections of the tissues were cut with a glass knife (5μm), placed on coated glass slides with a coverslip using Permount mounting medium (Fisher Scientific, SP15-500) and stained with Hematoxylin and Eosin (H&E) or Alcian blue (AB)-Periodic acid Schiff (PAS).

### Electron Microscopy (EM)

Immediately after removing the testis and EDs from the right side, each mouse was fixed by perfusion via the heart’s left ventricle with 2.5% glutaraldehyde in 0.1 M sodium cacodylate buffer, pH 7.4 containing 0.05% calcium chloride. After 10 min of perfusion, the left testis, efferent ducts, and epididymis were excised and cut into small 1 mm^3^ blocks, with samples taken of each of the 4 epididymal regions. All samples were placed in fresh fixative on ice for several hours and then washed overnight at 4°C in 0.1 M sodium cacodylate buffer (pH 7.4). The next day, samples were washed three times for 10 min each in cacodylate buffer and immersed for 2 hours in a solution containing 1% osmium tetroxide and 1.5% potassium ferrocyanide. All tissues were dehydrated in a graded series of acetone and embedded in Epon 812 medium (Mecalab Ltd., Montreal, QC). Thin sections (60 nm) of the testis, efferent ducts and 4 regions of the epididymis were cut with a diamond knife and mounted on copper grids for EM analysis and stained for 5 min with uranyl acetate and 3 min with lead citrate; grids were examined with a FEI Tecnai 12 120 kV Transmission Electron Microscope (TEM). Epon blocks of these tissues were also cut with glass knives (5 μm), placed on glass slides and stained with toluidine blue for LM analysis.

### Light microscope immunocytochemistry

Slides of paraffin sections (5μm) of testicular and epididymal tissues were deparaffinized in Citrisolv (Decon Laboratories Inc.) and rehydrated in a series of graded ethanol solutions. The sections were washed for 5 min in phosphate-buffered saline (PBS) and then immersed in a 7.1 mM citrate buffer (pH 6.0) and microwaved at full power for 3 min, followed by 7 min at 60% power. After cooling to room temperature, the slides were immersed in a peroxidase blocker solution containing 0.03% hydrogen peroxide and 0.031 M sodium azide (Dako Canada Inc., Mississauga, ON). They were then incubated overnight at 4°C with either anti-prosaposin (PSAP, RRID:AB_2792974) [79], anti-cytokeratin 5 (CK5, Covance Cat# PRB-160P-100, RRID:AB_10063444) [80] or anti-cathepsin D (CathD, Santa Cruz Biotechnology Cat# sc-6487, RRID:AB_637895) [81] antibodies in 50 mM Tris-Cl, pH 7.4 containing 1% Bovine serum albumin (BSA, Sigma A7511).

After incubation, the sections were washed six times for 5 min each with 0.1% Tween 20 in 50 mM Tris buffer, pH 7.4 containing 0.9% NaCl (TBST) and incubated for 60 min at room temperature with a secondary anti-goat (cat.401515, Calbiochem ® Burlington, MA, USA), anti-mouse (cat# A9044, Sigma, St. Louis, MO, USA) or anti-rabbit polymer-HRP (horseradish peroxidase) solution used directly from the kit (Dako EnVision+ System-HRP diaminobenzidine, Canada Inc., Mississauga, ON, Cat# K4010). The sections were then washed in TBST (5X, 2 min each) and treated with a 2% 3,3-diaminobenzidine (DAB) solution (Dako Canada Inc., Mississauga, ON). The sections were counterstained with methylene blue, dehydrated in a series of graded ethanol and Citrisolv, and mounted with coverslips using a Permount mounting medium. Negative controls were performed in the absence of the primary antibody.

### Quantitative analyses of normal versus abnormal seminiferous tubules

Seminiferous tubules of WT and KO mice were assessed qualitatively and considered normal when a full complement of germ and Sertoli cells was observed and a close affiliation of these cells with each. Cross sections of tubules were classified as abnormal when the epithelium was diminished in size, vacuolated and/or partially or entirely devoid of germ cells, i.e., when the diameter was approximately 50% or less of that noted for WT mice and which, at times resulted in the inability to stage the tubule.

H&E stained testis sections of 3 *Hgsnat+/+* and 3 *Hgsnat*−/− mice at 11 and 14 months of age were utilized with a range of 67–85 tubules counted for each animal. Photomicrographs were taken consecutively of cross sections of seminiferous tubules of a testis, moving the field from left to right, then top to bottom, thereby capturing tubules in a slide in the form of a cross. A mean percentage was generated for KO and WT mice and analyzed using an unpaired T-test for statistical analysis.

### Quantitative analyses of testicular and epididymal cross-sectional tubule profile areas

For this analysis, H&E stained epididymal sections of 3 *Hgsnat+/+* and 3 *Hgsnat*−/− mice were used at 11 and 14 months of age. Measurements of cross-sectional profile areas of seminiferous tubules of the testis and different regions of the epididymis were done using Version 4.5 of the Zeiss Axiovision LE Imaging Software (Carl Zeiss Canada Ltd., Toronto, ON). Digital images of H&E stained tubular cross sections were acquired with a Lumenera Infinity 1 camera (Lumenera Corp., Ottawa, ON). Sites for sampling cross-sectional profiles of the testis and epididymis were chosen consistently but randomly as mapped on a Cartesian plane. Selected tubules were outlined as follows; firstly, to define their tubule profile area (outer boundary area) and then to delineate their luminal profile area (inner boundary area). From these two measurements, the epithelial profile area (epithelial area) was deduced by subtraction (tubule profile area–Luminal profile area = epithelial area) as described in Parent et al. [82].

Measurements were in square micrometres. Statistical analyses were performed using Mann-Whitney tests (alternative nonparametric test) and GraphPad Prism software (GraphPad Software Inc., USA) with p values less than 0.05 were considered significant.

### Sperm motility

Cauda epididymides of 5 *Hgsnat+/+* and 8 *Hgsnat*−/− mice at 11 months were submerged in Medium 199, Hanks’ Balanced Salts (Gibco, cat# 12350–039). Epididymides were cut and squeezed gently to allow spermatozoa to diffuse into suspension for 10 min at 37°C. Sperm concentration was determined with a hemocytometer and then adjusted to 5 × 10^6^ cells/ml in Medium 199, Hanks’ Balanced Salts. Sperm motility parameters were determined using a computer-assisted sperm analysis system (CASA) with Sperm Vision HR software version 1.01 (Minitube, Ingersoll, ON, Canada) [83]. Two hundred spermatozoa were examined for each sample to determine each sperm motility parameter. Mann-Whitney tests (alternative nonparametric test) were done using Version 8 of Statistica for Windows (Statsoft, Inc., Tulsa, OK). The p values less than 0.05 were considered significant.

### Scanning electron microscopy (SEM) of sperm of *Hgsnat+/+* and *Hgsnat*−/− mice

Spermatozoa from *3 Hgsnat+/+* and 3 *Hgsnat*−/− 11-month old male mice were squeezed out of freshly isolated mouse cauda epididymides in Hanks’ Balanced Salts using sterile forceps and allowed to ‘swim out’ for 10 min at 37°C. Coverslips coated with 0.1% polylysine were used to incubate cauda spermatozoa for 20 min. Sperm fixation was performed with 4% paraformaldehyde for 1 hour. Samples were covered with 3 nm of platinum at room temperature and visualized at high vacuum by a FEI Quanta FEG 450 Scanning Electron Microscope.

### Transmission electron microscopic (TEM) evaluation of sperm of *Hgsnat+/+* and *Hgsnat*−/− mice

Eleven selected frames of TEM images of the lumen of cauda epididymides were analyzed from adult mice (*3 Hgsnat+/+* and 3 *Hgsnat*−/−) 11 months of age. For each frame (area unit: 245.5 μm^2^), the number of abnormal sperm tails (abnormally bent 90 degrees or greater along their long axis) and total sperm heads were counted in WT and KO mice. Statistical differences between groups were determined using student test analysis, and differences among samples were considered significant with a p-value of less than 0.05.

### *In vitro* fertilization

Cumulus-oocyte complexes from superovulated C57Bl6 mice were collected by piercing oviducts placed in human tubal fluid (HTF; Millipore catalogue no. MR-070-D) 20 h after hCG injection. Oocytes were immediately inseminated after collection, as described by Ferrer et al. [84]. Spermatozoa from an adult male (3 *Hgsnat+/+* and 3 *Hgsnat*−/−) mice 7 month-old were squeezed out of freshly isolated mouse cauda epididymides in HTF media using sterile forceps and allowed to ‘swim out’ for 10 min at 37°C. Sperm were counted using a hemocytometer and inseminated with an equal number of oocytes. Insemination and subsequent wash and rest steps occurred in 50 μl HTF drops overlaid with sterile mineral oil in a culture dish. Cumulus-oocyte complexes were inseminated with 10^6^ sperm/ml in HTF. After 20 h incubation (37°C, 5% CO2), oocytes successfully fertilized were assessed by the presence of a second polar body or cleavage. Trials for each group were repeated at least three times. A chi-square homogeneity test with the Bonferroni correction was performed to assess the significant difference between the groups.

## Results

### Effects of *Hgsnat* gene inactivation on the testis

**Fig 1:**
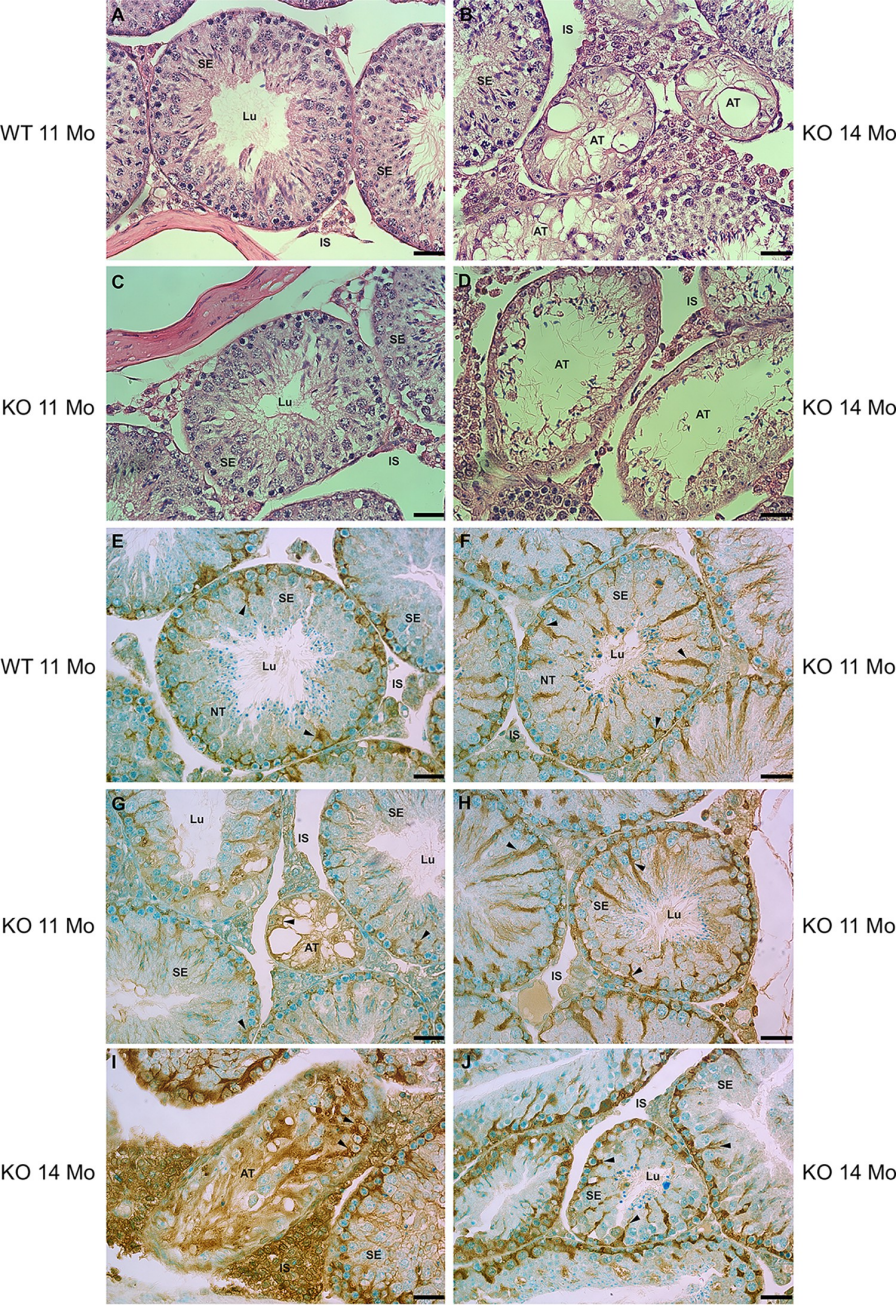
LM of seminiferous epithelium (SE) of the testis stained with H&E (A-D) and anti-prosaposin antibody (E-J) of WT (A, E) and KO (B-D, F-J) mice at 11 or 14 months of age. In (F), several KO tubules demonstrate a normal appearance (NT) as seen in WT (A), while other KO tubules appear abnormal (AT) with a disrupted epithelium (B, D, G and I); some KO tubules only reveal Sertoli cells (B, G). Sertoli cells (arrowheads) immunolabeled for prosaposin are evident in WT (E) and KO (F-J) mice, as well as in the grossly altered tubules (G, I). Lu, lumen; IS, interstitial space. Scale bars = 35 μm.

*Hgsnat*-/- and *Hgsnat+/+* mice were examined at 7, 11 and 14 months of age. LSD develops with time due to the progressive accumulation of undigested substrate in the lysosomes. In this case, significant structural alterations of both the testis and epididymis were noted at 11 and 14 months of age. Therefore, we focused on these age groups only. Seminiferous tubules of the *Hgsnat*-/- mice testis demonstrated a diversity of structural abnormalities in KO compared to WT mice, as shown by LM analysis of H&E-stained tissues (Fig 1A–1D) and seminiferous tubules labeled with anti-prosaposin antibodies (Fig 1E–1J). The latter demonstrated reactive Sertoli cells [79] with a radial spoke-like appearance in WT and KO animals (Fig 1E–1J). In KO mice (Fig 1F), some tubules had a normal complement of germ cells enveloped by Sertoli cells, with a morphology comparable to WT mice (Fig 1A and 1E). However, other tubules appeared to be adversely affected and demonstrated a partial (Fig 1I and 1J) or complete absence of germ cells and revealing a highly vacuolated appearance and a dramatic reduction in tubular profile area (Fig 1B–1D, 1G and 1H).

**Fig 2:**
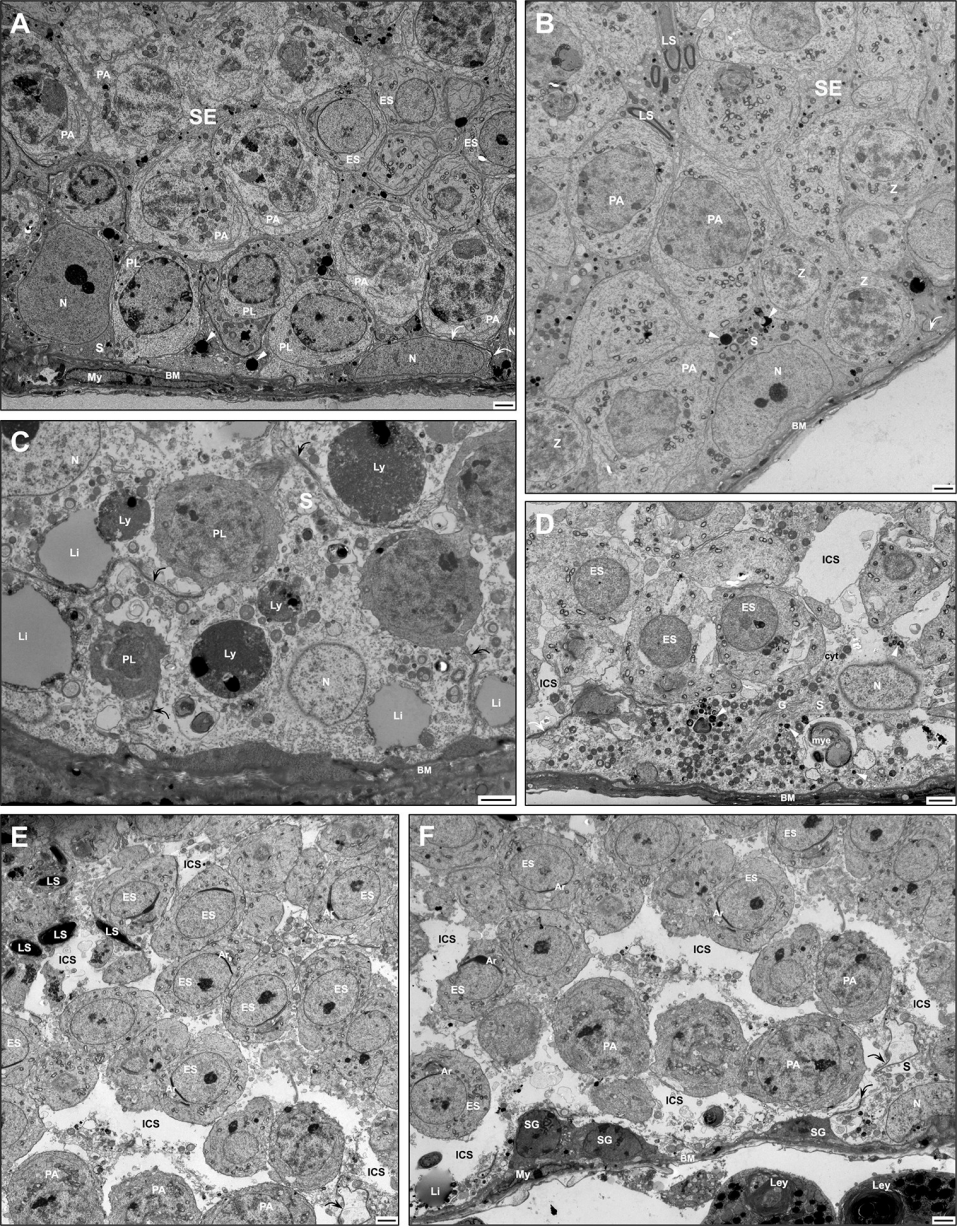
EM of seminiferous epithelium (SE) of WT (A, B) and KO (C-F) KO tubules at 11 or 14 months of age. In (A, B), tubules reveal a close approximation of germ cells with Sertoli cells (S), small dense lysosomes (arrowheads) and intact BTB (curved arrows). In (C), abnormally large and medium-sized dense granulated lysosomes (Ly) are prominent in SE, as well as large lipid droplets (Li). In (D), numerous small lysosomes (arrowheads) occupy the base of Sertoli cells, along with a large myelinated-like body (mye). In (C), Sertoli cells reveal an intact BTB (curved arrows), while in (D-F), it is disrupted. In (D-F), Sertoli cells show abnormal bloated basal and adluminal compartments resulting in dilated intercellular spaces (ICS) confluent with the cytoplasm (cyt) and containing organelles and membranous profiles. Germ cells: spermatogonia (SG), early (ES) and late (LS) spermatids; pachytene (PA) and preleptotene (PL) spermatocytes; zygotene (Z) spermatocytes; As, acrosome; BM, basement membrane; My, myoid cells; N, Sertoli cell nucleus. Scale bars = 2 μm.

EM analysis also showed a variety of structural alterations to germ and Sertoli cells (Fig 2C–2F, S1A–S1D Fig) compared to WT animals (Fig 2A and 2B). In WT tubules, small spherical electron-dense lysosomes were noted at the base of Sertoli cells of the seminiferous epithelium (Fig 2A and 2B), while in KO tubules, considerably larger lysosomes presented a varying phenotype (Figs 2C, 2D, S1A and S1D) including large spherical lysosomes with a granular dense content (Fig 2C, S1D Fig). In some KO tubules, homogeneous electron-dense lysosomes of varying sizes occupied the base with smaller lysosomes appearing to fuse with the larger ones (S1A Fig). Large lipid droplets with a pale homogeneous appearance often shared the cytoplasm with these lysosomes (Fig 2C). Several abnormal more-or- less spherical bodies of moderate size were also observed at the base of Sertoli cells of KO tubules with a myelinated central mass enveloped by a pale stained corona (Fig 2D and 2E).

In some KO tubules, an absence of Sertoli cell processes enveloping early and late germ cells was noted, which appeared to be due to the disruption of Sertoli cells (Fig 2D–2F; S1B Fig). On occasion, the Sertoli-Sertoli barrier, constituting the blood-testis barrier (BTB), was discontinuous or ruptured along its length suggestive of a breakdown of adjacent Sertoli cell membranes at this site (Fig 2F, S1C Fig) resulted in cellular organelles appearing to leak out of their cytoplasm (Fig 2D; S1B and S1D Fig). Such tubules revealed grossly swollen intercellular spaces that contained membranous profiles of different sizes, granulated bodies and organelles, some characteristic of Sertoli cells (mitochondria, small lysosomes, lipid droplets) (Fig 2F; S1A–S1D Fig). Moreover, the nuclei of Sertoli cells in some tubules were erratic in their distribution and position (Fig 2D and 2E; S1A and S1D Fig), with some being basally located while others distributed higher up in the epithelium and clustered together (S1D Fig).

In some KO tubules, abnormal vacuolated areas of the cytoplasm were also noted in spermatocytes and early spermatids appearing as washed-out areas (S1A Fig). However, even in grossly altered tubules, spermatogonia were evident at the base of the epithelium (Fig 2F, S1B Fig). Despite the presence of washed-out areas of cytoplasm in early spermatids, the Golgi apparatus and acrosome formation were intact (Fig 2E and 2F, S1B Fig). Overall, Leydig cells of the interstitial space of KO mice (S1B Fig) were comparable in appearance to that of WT animals. Although the absence of the *Hgsnat* gene did have significant effects on some tubules and Sertoli cells, not all tubules were adversely compromised. Such an on/off phenomenon of normal alongside affected tubules in the testis has been noted for several different KO mouse models, as discussed below. Unlike other lysosomal storage disorders, the absence of HGSNAT did not affect the morphology of interstitial macrophages [51].

A qualitative assessment of normal versus abnormal seminiferous tubules of H&E stained sections of WT and KO mice revealed values ranging from 1–3% in WT up to 7–18% in KO mice (mean value approximately 12%) with values being statistically significant (p<0.0374) from each other (S2A Fig).

Values of luminal, epithelial and tubular profile areas (μm^2^) were also calculated at the early and late stages of the WT and KO mice cycle. Stages VI-VIII (S2B Fig) represented early stages containing 2 generations of spermatids with relatively high duration rates, and stages IX -XII (S2C Fig) as late stages with a single generation of late spermatids and lower duration rates. In each case and at both age groups, there were significant statistical differences in the tubule and epithelial profile areas as compared with WT mice but not in the luminal profile area, suggesting alterations to the seminiferous epithelium consisting of germ and Sertoli cells (S2B and S2C Fig).

### Effects of *Hgsnat* gene inactivation on the efferent ducts and epididymis

**Fig 3:**
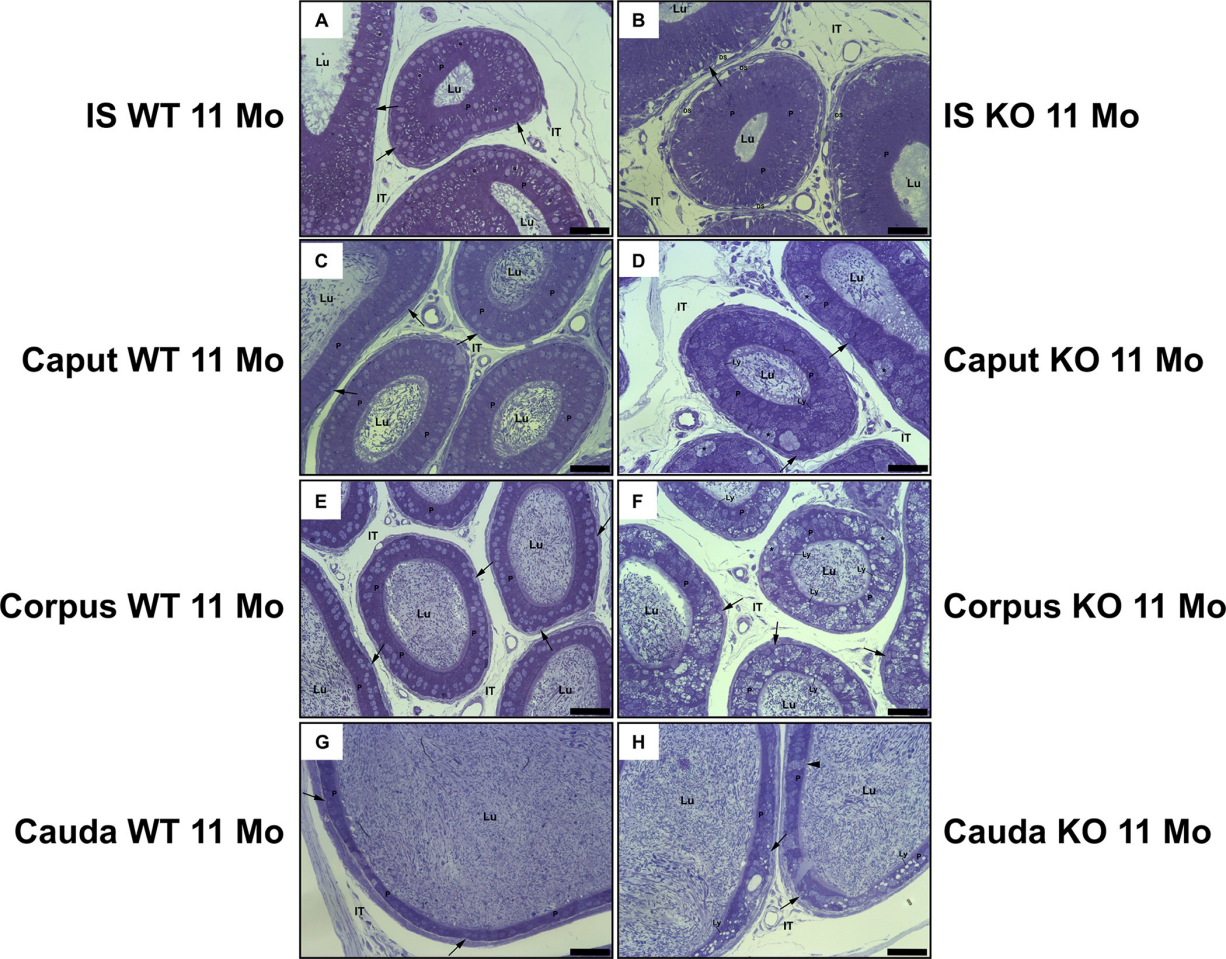
Toluidine blue stained sections of different regions of the epididymis of WT (A, C, E, G) and KO (B, D, F, H) mice. In (B), principal cells (P) of the initial segment (IS) of KO mice appear taller in appearance compared to WT mice (A), and large elongated dilated spaces (DS) occupy the base of the epithelium (B). In the caput, corpus and cauda regions of KO mice (D, F, H), a plethora of small to large-sized pale lysosomes (large arrows) occupy the cytoplasm of principal cells (P), which are not noted in WT cells (C, E, G). In addition, very large pale stained foamy eMPs (asterisks) converge at the base of the epithelium of KO mice of the caput and corpus regions (D, F), which are not noted in WT mice (C, E). The lumen (Lu) of KO mice reveals an abundance of sperm. Basal cells, small arrows; IT, intertubular space. Scale bars = 50 μm.

In toluidine, blue stained sections of 11 and 14-month-old mice, the epithelium of the initial segment revealed tall columnar principal cells that appeared larger in KO (Fig 3B) than WT (Fig 3A) mice. Moreover, elongated dilated intercellular spaces occupied the base of KO tubules (Fig 3B) that were not observed in WT mice (Fig 3A). In KO mice of the caput (Fig 3D) and cauda (Fig 3H) regions, principal cells contained numerous small to large pale stained lysosomes not evident in their WT counterpart (Fig 3C, 3G). In the corpus of KO mice (Fig 3F), the accumulation of pale lysosomes was more prominent than in the caput and cauda regions (Fig 3E).

In all regions, sporadic large, more or less spherical cells with a foamy appearance were lodged at the base of the epithelium of KO mice; they occupied a sizeable area of the basal epithelium, contained a flocculent material and rarely demonstrated an association with the lumen (Fig 3B, 3D, 3F and 3H). For reasons noted below they were referred to as eMPs.

In LM of PAS-stained sections, the efferent ducts of WT animals were small with a simple columnar epithelium of resident ciliated and nonciliated cells (S3A Fig). In KO mice, the diameter of the tubules appeared enlarged in size (S3B Fig). The diameter of the tubules of the corpus region also appeared to be larger in KO (S3D Fig) as compared to WT mice (S3C Fig). As demonstrated with toluidine blue sections, several eMPs cells of the corpus region of KO mice were noted at the base of the epithelium (S3D and S3E Fig) as well as enlarged clear cells in the cauda region (S3F Fig).

The mean tubule, luminal and epithelial profile areas of the initial segment, caput, proximal and middle corpus regions were evaluated and graphed in S4 Fig. In the initial segment, the mean luminal profile area significantly decreased in KO mice compared to WT mice (P value of < = 0.001). Conversely, in the caput and proximal corpus, the mean tubule profile and epithelial area significantly increased with P values of < = 0.01 for the tubule profile of caput and P < = 0.001 for the rest of the mentioned areas. The luminal profile areas (tubular area-epithelial area) of the caput and proximal corpus regions were not statistically different (P > 0.05) between KO and WT mice. In the middle corpus region, the luminal profile area showed a significant increase (P < = 0.05) in KO mice. In contrast, the mean tubular and epithelial profile areas revealed highly significant increases (P < = 0.001) compared to the wild type (S4 Fig).

**Fig 4:**
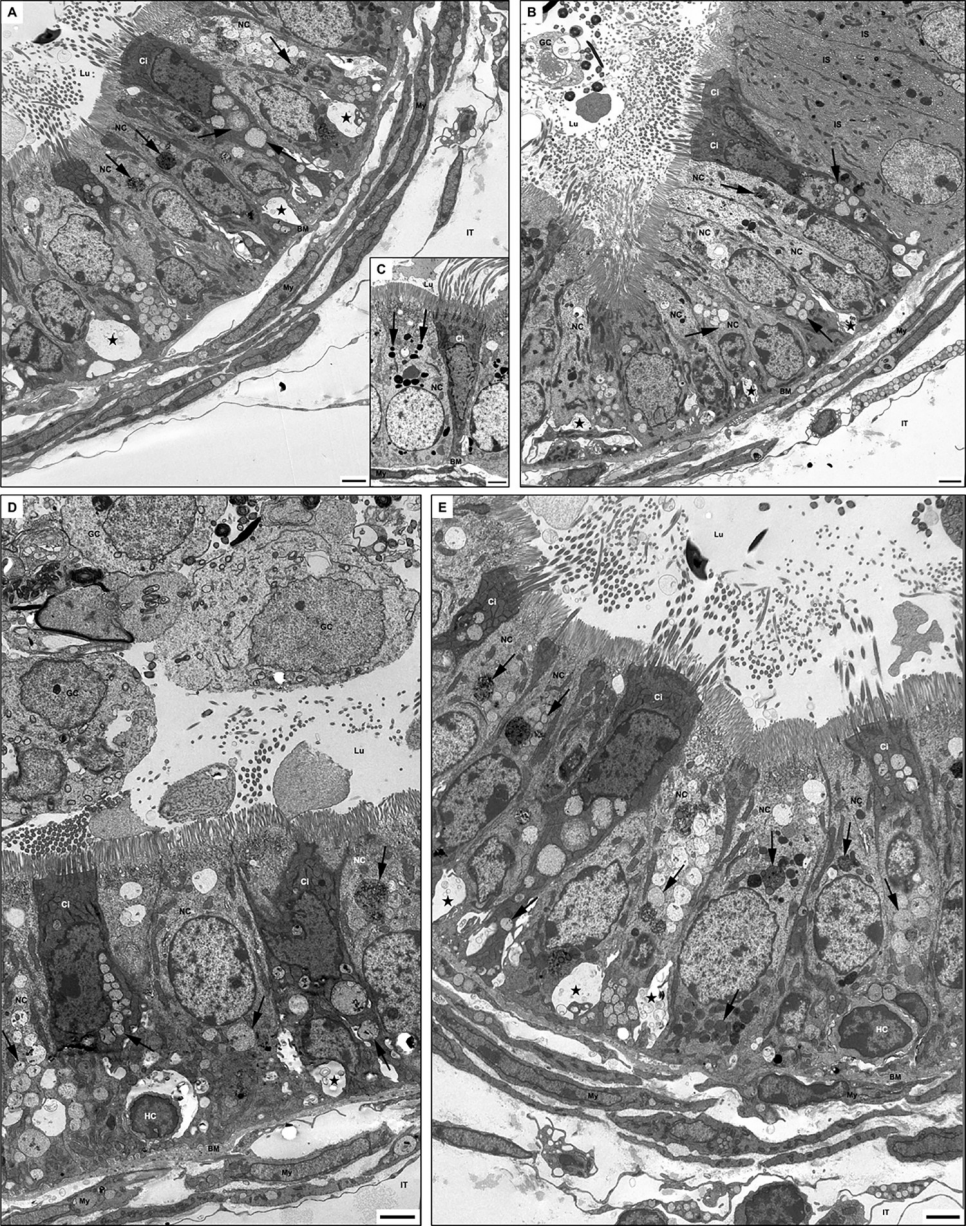
EM of efferent ducts of WT type (C) and KO (A, B, D, E) mice. In (C), a few small dense lysosomes (arrows) appear in nonciliated (NC) and ciliated (Ci) cells. At the same time, KO mice reveal medium to large-sized pale stained and dense lysosomes supra and infranuclearly (A, B, D, E). In (B), a small portion of the initial segment (IS) is observed. Dilated intercellular spaces (stars) containing membranous profiles are evident at the base of the epithelium of KO mice (A, B, D, E). In (D), germ cells (GC) appear in the lumen. In (D, E), halo cells (HC) are seen at the base of the epithelium. BM, basement membrane; My, myoid cells; Lu, lumen; IT, intertubular space. Scale bars = 2 μm.

As noted by EM, the small electron-dense supranuclear lysosomes of nonciliated cells of WT mice (Fig 4C) were replaced in KO mice by numerous pale stained lysosomes of different sizes lodged in the supra and infranuclear areas of their cytoplasm (Fig 4A, 4B, 4D and 4E). Such lysosomes contained loose membranous profiles and small vesicles embedded in a flocculent material. At times, a few large lysosomes, dense in appearance and containing an electron-dense granule-fibrillar material, were also noted in the cytoplasm (Fig 4A and 4E). The deeply stained ciliated cells revealed numerous small and larger pale lysosomes in their supra and infranuclear cytoplasm containing a flocculent material and granular debris (Fig 4A, 4B, 4D and 4E), which were not a feature of WT mice (Fig 4C).

Notably, the intercellular space at the base of the epithelium was often dilated and contained membranous profiles of varying shapes and sizes. The basement membrane was thickened and multilayered, and myoid cells and fibrocytes of the tunica propria also contained large pale stained lysosomes (Fig 4A, 4B, 4D and 4E). Some immature germ cells resided in the lumen, suggesting their sloughing off from the seminiferous tubules of the testis (Fig 4D). Halo cells were noted at the base of the epithelium but with a normal appearance (Fig 4D and 4E).

### EM ultrastructural appearance of the epididymis of WT and KO mice

**Fig 5:**
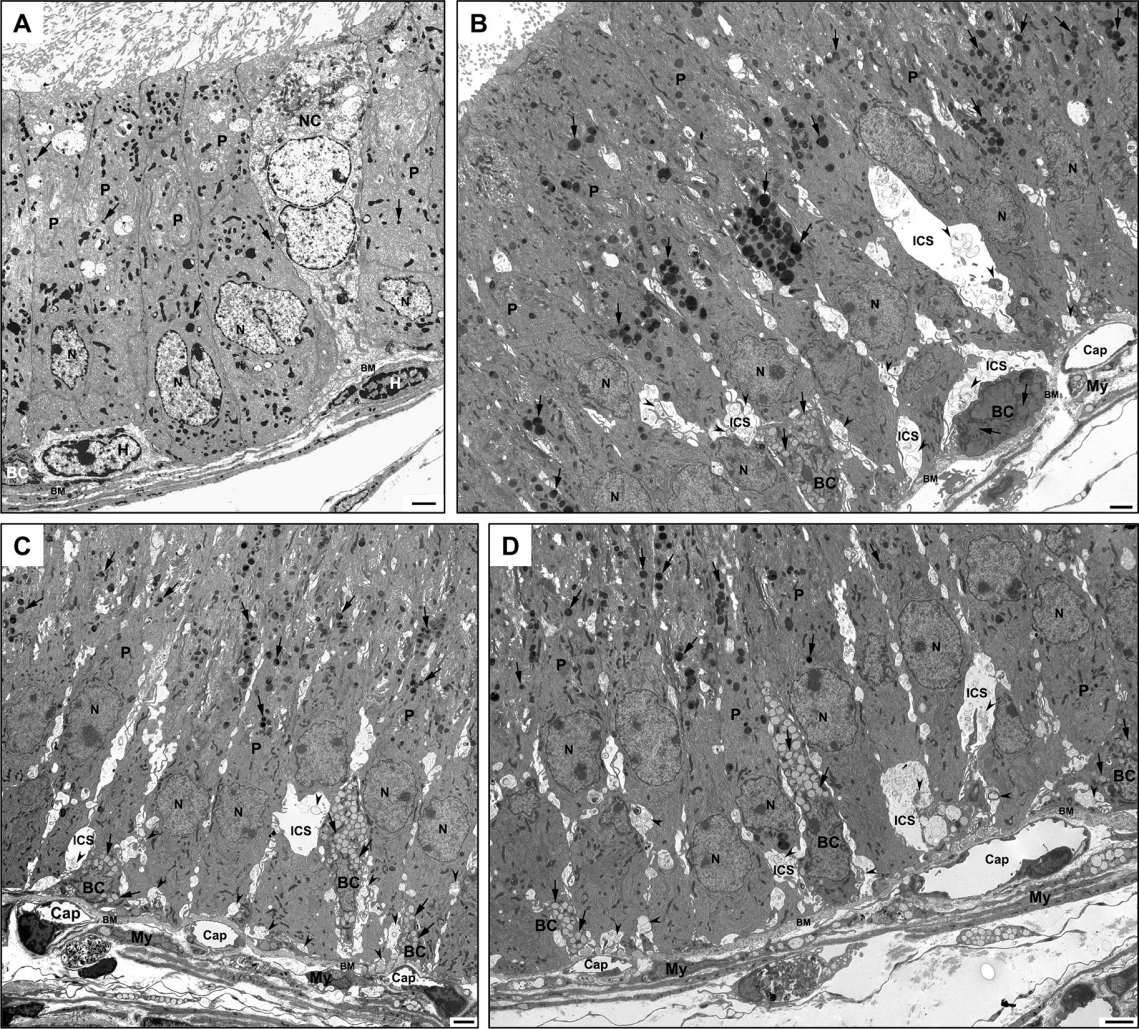
EM of the initial segment of WT (A) and KO (B-D) mice. In (A), tall columnar principal cells (P) reveal a few small dense lysosomes (arrows). A halo cell (H) is adjacent to a small nondescript basal cell (BC), and a large narrow cell (NC) is noted. In KO mice, the dense lysosomes (arrows) appear to be more abundant and larger in size (B-D) as compared to WT mice (A). In (B-D), basal cells of KO mice reveal different shapes, sizes and commitment to the basement membrane and are filled with small to medium-sized pale lysosomes (arrows). Basally located dilated intercellular spaces (ICS) of KO mice contain membranous and vesicular profiles (arrowheads) (B-D). Cap, capillaries; My, myoid cells; BM, basement membrane; N, nucleus. Scale bars = 2 μm.

In the epididymis, principal cells of the initial segment (IS) of 11 and 14-month-old KO mice were modified but not to the degree of that noted in the other regions. In the IS, the lysosomes of WT mice were small and spherical and few in number scattered in the supranuclear area of the cytoplasm (Fig 5A). Large multivesicular bodies were also noted amongst the small dense lysosomes (Fig 5A). In KO mice, lysosomes of some principal cells appeared to be more abundant, with some of larger size. They occasionally clustered together while retaining their supranuclear position and dense appearance (Fig 5B–5D). The other organelles of the cytoplasm, such as the Golgi apparatus, mitochondria and ER cisternae, appeared comparable to WT mice.

Notably, intercellular spaces of the initial segment of KO mice became prominent as they were highly dilated and dominated the lower half of the epithelium (Fig 5B–5D). As a result, the cytoplasmic finger-like processes formed at the lateral plasma membrane of adjacent principal cells were highlighted as they dangled freely in the dilated spaces (Fig 5B–5D). The lack of association of adjacent processes appeared to affect the adhesion properties between principal cells, often resulting in only small focal contact points. The formation of extensive intercellular dilated spaces also compromised the adhesion of principal cells to the basement membrane, with some contacting only by small foot-like processes (Fig 5B–5D). The dilated spaces were loaded with membranous profiles and vesicles of varying sizes and shapes (Fig 5B–5D), suggesting abnormal alteration to the epithelium.

Strikingly, while the basal cells of WT mice were small, with a lackluster cytoplasm and firm adherence to the basement membrane (Fig 5A), in KO mice, they were dramatically different in size and appearance (Fig 5B–5D). Overall, basal cells of KO mice contained numerous small to medium-sized pale stained lysosomes (Fig 5B–5D). While some basal cells of KO mice were rounded and plumper with a well-defined adherence to the basement membrane, others were taller slender, elongated cells orientating themselves toward the lumen and filled with pale lysosomes (Fig 5B–5D). The dilated spaces allowed basal cells to be seen to advantage, with some revealing little to no apparent contact with the basement membrane in given planes of section, but identifiable by their size, shape and location at the base of the epithelium and plethora of small pale lysosomes (Fig 5B–5D).

The lamina propria underlying the epithelium contained subepithelial capillaries in the proximal initial segment as previously described in WT mice [85] and as noted for this region in KO mice (Fig 5B–5D). Unlike WT mice, the basement membrane was considerably thickened in KO mice and myoid cells formed concentric layers around the epididymal tubules and, at times, demonstrated numerous pale lysosomes (Fig 5B–5D).

**Fig 6:**
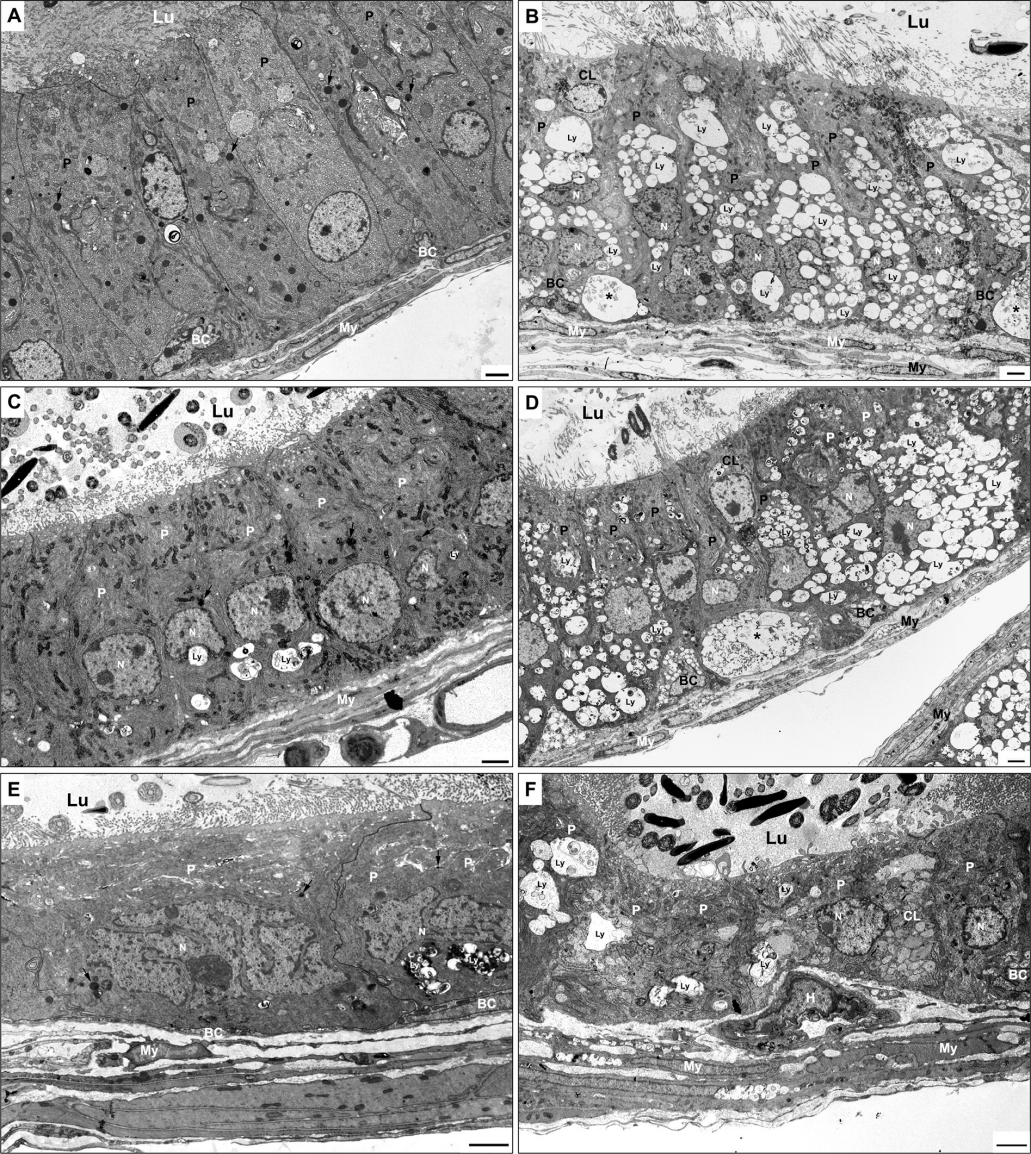
EM of caput (A, B), corpus (C, D) and cauda (E, F) regions of WT (A, C, E) and KO (B, D, F) mice. In WT mice (A, C, E), principal cells (P) reveal few small dense supranuclear lysosomes (arrows) and occasional irregular larger dense lysosomes (Ly) infranuclearly (C, E). In all 3 regions of KO mice, principal cells (P) exhibit a plethora of medium to large-sized pale lysosomes (Ly) in their supra-and infranuclear areas (B, D, F), Clear cells (CL) are evident in KO mice (B, D, F) and at times show numerous pale stained lysosomes (F). Basal cells (BC) of KO mice (B, D, F) show prominent small to moderate pale stained lysosomes, which are not noted in WT mice (A). Large basally located eMPs (asterisks) reside in KO mice at the base of the epithelium (B, D). My, myoid cells; Lu, lumen; N, nucleus; H, halo cell. Scale bars = 2 μm.

Unlike the initial segment, the caput, corpus and cauda regions demonstrated dramatic morphological differences with regard to principal cells in both 11 and 14-month-old KO mice. In all 3 regions of WT mice, few small dense spherical lysosomes were observed in the supranuclear cytoplasm of principal cells (Fig 6A, 6C and 6E), with only sporadic larger, more irregular lysosomes noted in the infranuclear cytoplasm of the corpus and cauda regions (Fig 6C and 6E). In KO mice, a plethora of pale lysosomes of different shapes and sizes occupied the supra- and infranuclear cytoplasm of principal cells of all 3 regions (Fig 6B, 6D and 6F; S5A–S5D and S6A–S6D Figs). Pale lysosomes of smaller size appeared to be fusing to form larger pale lysosomes and which accounted for some of the considerable size; they contained membranous profiles and a flocculent material (Fig 6B, 6D and 6F; S5A–S5D and S6A Figs).

In KO mice, clear cells of KO mice looked either normal in appearance or engorged with large pale lysosomes (Fig 6B, 6D and 6F, S5A, S5C, S5D and S6B Figs). Unlike the small hemispherical or elongated basal cells adhering to the basement membrane of the caput, corpus and cauda regions of WT mice (Fig 6A), those of KO mice were larger in size and contained numerous small to moderate-sized pale lysosomes (Fig 6B, 6D and 6F; S5A, S5C and S5D Fig). Intriguingly, at the base of the epithelium of the entire epididymis of KO mice, eMPs were prominent (Fig 6B and 6D). Myoid cells of these regions were at times affected and showed numerous pale lysosomes (Fig 6D and 6F; S5A and S5B Fig).

**Fig 7:**
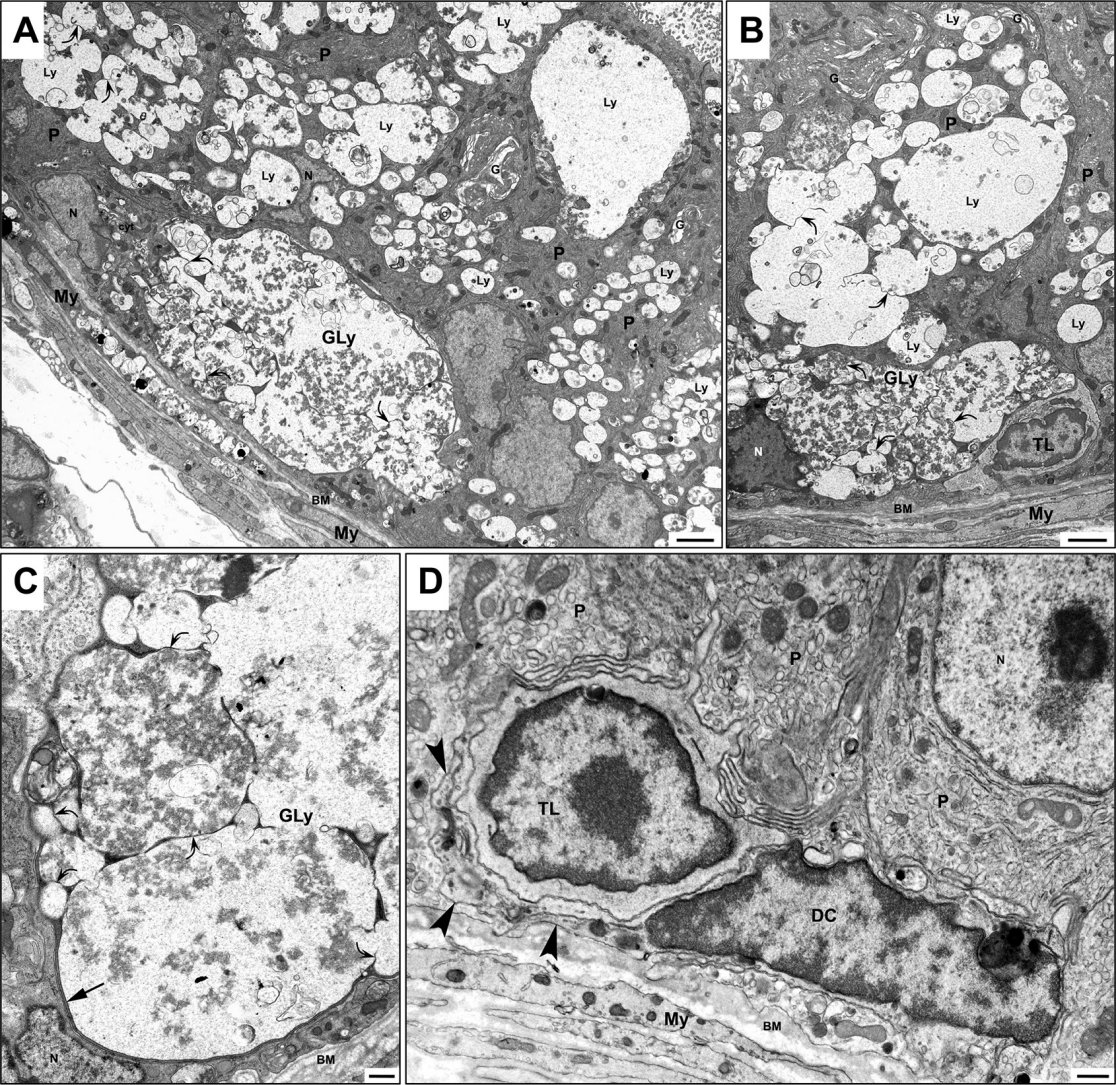
EM of KO (A-C) and WT (D) mice. In (A-C), large eMPs straddle the basement membrane and reveal a cytoplasm dominated by a single gigantic pale stained lysosome (Gly) containing numerous membranous profiles and finely dense granular material. Invaginations of the plasma membrane of Gly appear as thin needle-like projections (curved arrows) that protrude deep into the Gly and, at times, branch but do not penetrate its interior. During the formation of the Gly by fusion of smaller lysosomes with each other, these projections trap the cytoplasmic matrix (A-C) as the Gly greatly increases in size and excludes organelles leaving only a thin layer of deeply stained homogeneous material between the entrapped invaginated plasma membrane of the Gly. Principal cells (P) demonstrate numerous pale stained lysosomes (Ly) of different shapes and sizes and contain membranous profiles. Notably, their large lysosomes also reveal thin invaginations (curved arrows) of their plasma membrane, which extend deep into its interior and likewise trap the cytoplasmic matrix excluding organelles. In WT mice (D), a dendritic cell (DC) reveals a thin process (arrowheads) which appears to wrap itself around a halo cell identified as a T lymphocyte (TL). In (B), a small spherical T-lymphocyte (TL) is adjacent to an eMP cell, suggested to be a dendritic cell. BM, basement membrane; N, nucleus; My, myoid cells. Scale bars = A and B = 2 μm, and C and D = 500 nm.

By EM, the striking features of the eMPs in KO mice were their large size, pale stained appearance, predominant residence in the lower half of the epithelium and sporadic commitment to the basement membrane (Fig 7A–7C). The nucleus of the eMPs was small and densely stained and occupied one corner of the cell where the only existing and sparse organelles hovered in the remaining bit of cytoplasm (Fig 7A and 7B). Detailed analysis of these cells revealed that a gigantic lysosome took full occupancy of their cytoplasm (Fig 7A–7C). The giant lysosome appeared as a large membrane-bound organelle revealing numerous invaginations of its surface membrane. It formed thin needle-like processes that penetrated its interior but did not pierce it, like fingers into a balloon. Overall, the invaginations of the surface projections imparted a notched appearance to the gigantic lysosome. It is suggested that following endocytosis of substances by the eMPs, lysosomes are formed, which in the absence of HGSNAT, cannot break down the internalized substances. As a result, the smaller lysosomes fuse with each other and gradually form the gigantic lysosome. As they do so, areas of the cytoplasmic matrix become trapped and squeezed by the closely apposed invaginating membranes, excluding organelles other than a darkly stained homogeneous cytoplasmic matrix (Fig 7A–7C). Contents of the gigantic lysosome included irregularly shaped membranous profiles of different shapes and sizes and patches of moderately dense granule-vesicular debris (Fig 7A–7C). Confirmation of the formation of the gigantic lysosomes of the eMPs came from images of the large pale lysosomes noted in principal cells of KO mice. In these cells, the large lysosomes were notched by thin, slender invaginations that likewise trapped and squeezed areas of the cytoplasmic matrix (Fig 7B). Such lysosomes contained membranous profiles of different shapes and sizes and granule-vesicular debris (Fig 7A–7C).

Interestingly, an occasional feature of some eMPs was their close association with a population of halo cells, identified as T-lymphocytes (Fig 7B) from early studies [68]. Indeed, in many body tissues, T-lymphocytes have been described to cross-talk with dendritic cells [86–88]. In our analysis of WT mice, T-lymphocytes associated with a population of slender cells residing at the base of the epithelium and that closely enveloped T-lymphocytes by their thin processes; the latter may represent the resident dendritic population of cells as noted in KO (Fig 7B) and WT (Fig 7D) mice, a topic to be discussed below.

### LM immunostaining of epididymis of WT and KO mice with anti-cytokeratin 5 (CK5), anti-prosaposin (PSAP) and anti-Cathepsin D (CathD) antibodies

**Fig 8:**
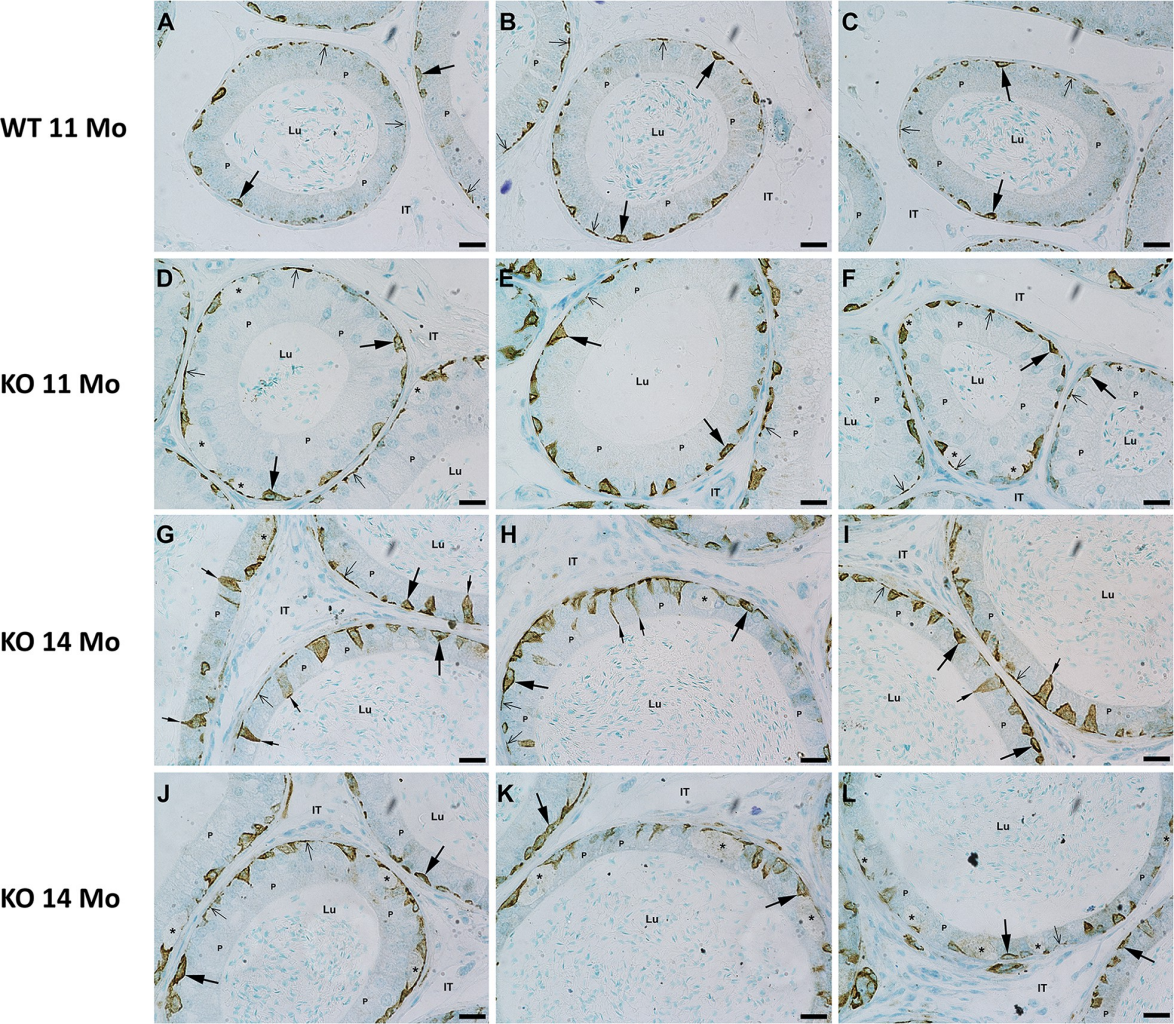
LM immune of the epididymis of WT (A-C) and KO (D-L) mice with anti-CK5 antibody. Basal cells (large arrows) are intensely reactive. However, the eMPs are unreactive (asterisks). Some basal cells contact the lumen (small arrows). Isolated processes of basal cells hug the basement membrane (small thin arrows). Lu, lumen; IT, intertubular space; P, principal cells. Scale bars = 20 μm.

LM immunostaining of epididymal sections of WT mice at 11 and 14 months of age with anti-CK5 antibody revealed intense reactions over basal cells of WT mice (Fig 8A–8C), for which CK5 has been assigned as a specific marker [71, 72, 89]. Some WT basal cells were small and hemispherical, while others were flattened and elongated (Fig 8A–8C). In KO mice, basal cells maintained their reactivity and appeared larger and more prominent, at times, with a triangular appearance (Fig 8A–8L). Some basal cells contacted the lumen (Fig 8G–8I), while others sent thin processes upward toward the lumen (Fig 8G–8L). In WT and KO mice, lateral processes extended from the main central body of basal cells along the basement membrane. Some discrete, isolated focal reactions were noted and presumed to be sections through these processes. Interestingly, no reaction was observed over the large pale stained basally located eMPs of KO mice (Fig 8D, 8H, 8J and 8K).

**Fig 9:**
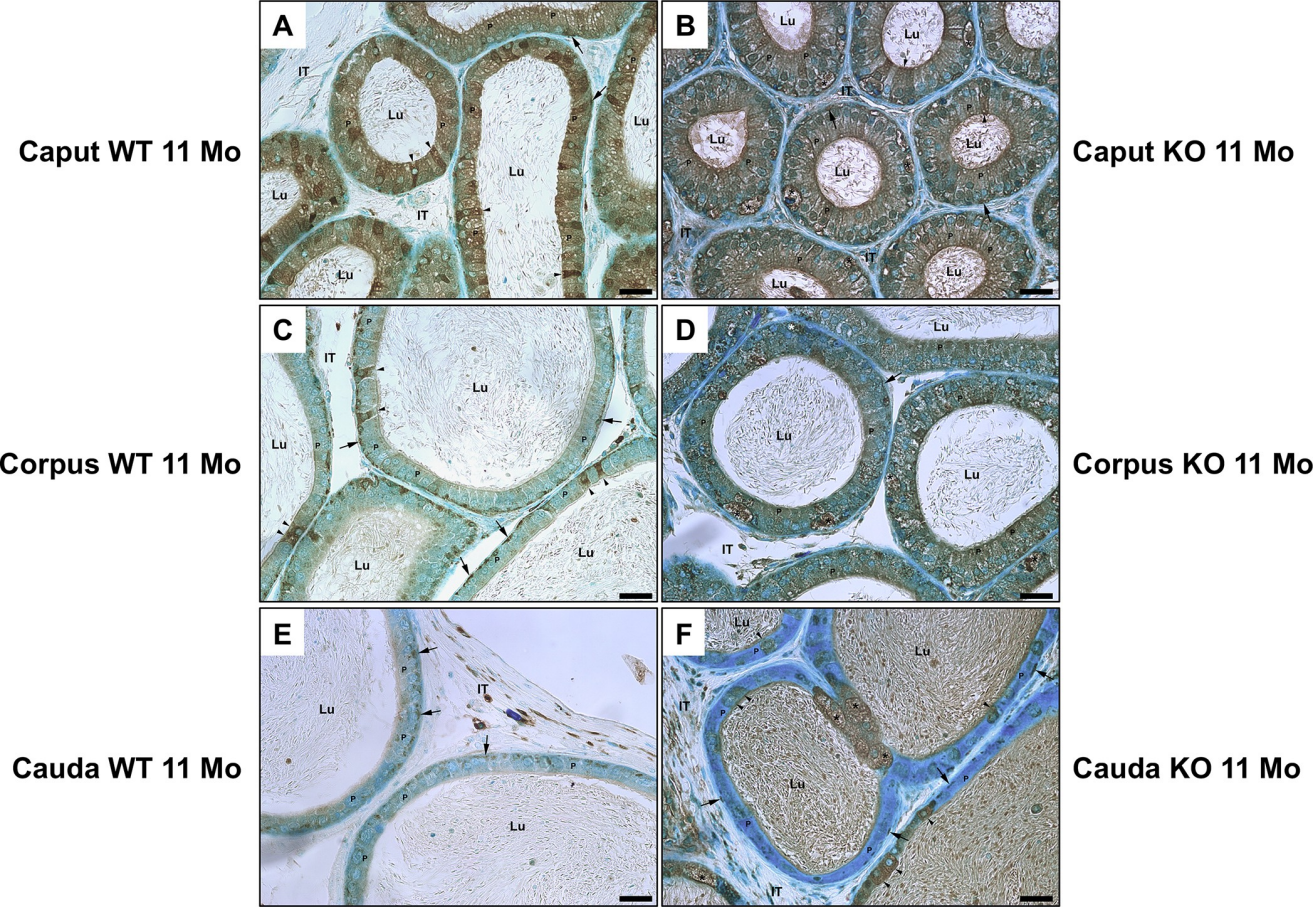
LM immune reaction of the epididymis of WT (A, C, E) and KO (B, D, F) mice with anti-PSAP. Intense reactions appear over large, dilated eMPs (asterisks) at the base of the epithelium of KO mice which are not prominent in WT mice. Basal cells are reactive (arrows), as well as principal cells (P) and clear cells (arrowheads). Lu, lumen; IT, intertubular space. Scale bars = 20 μm.

LM immunocytochemical staining of epididymal sections of KO mice at 11 and 14 months of age with anti-PSAP antibody revealed an intense reaction over the large pale stained eMPs (asterisks) lodged at the base of the epithelium of the caput, corpus and cauda regions of KO mice (Fig 9B, 9D and 9F). Such cells were not prominent in WT mice (Fig 9A, 9C and 9E).

**Fig 10:**
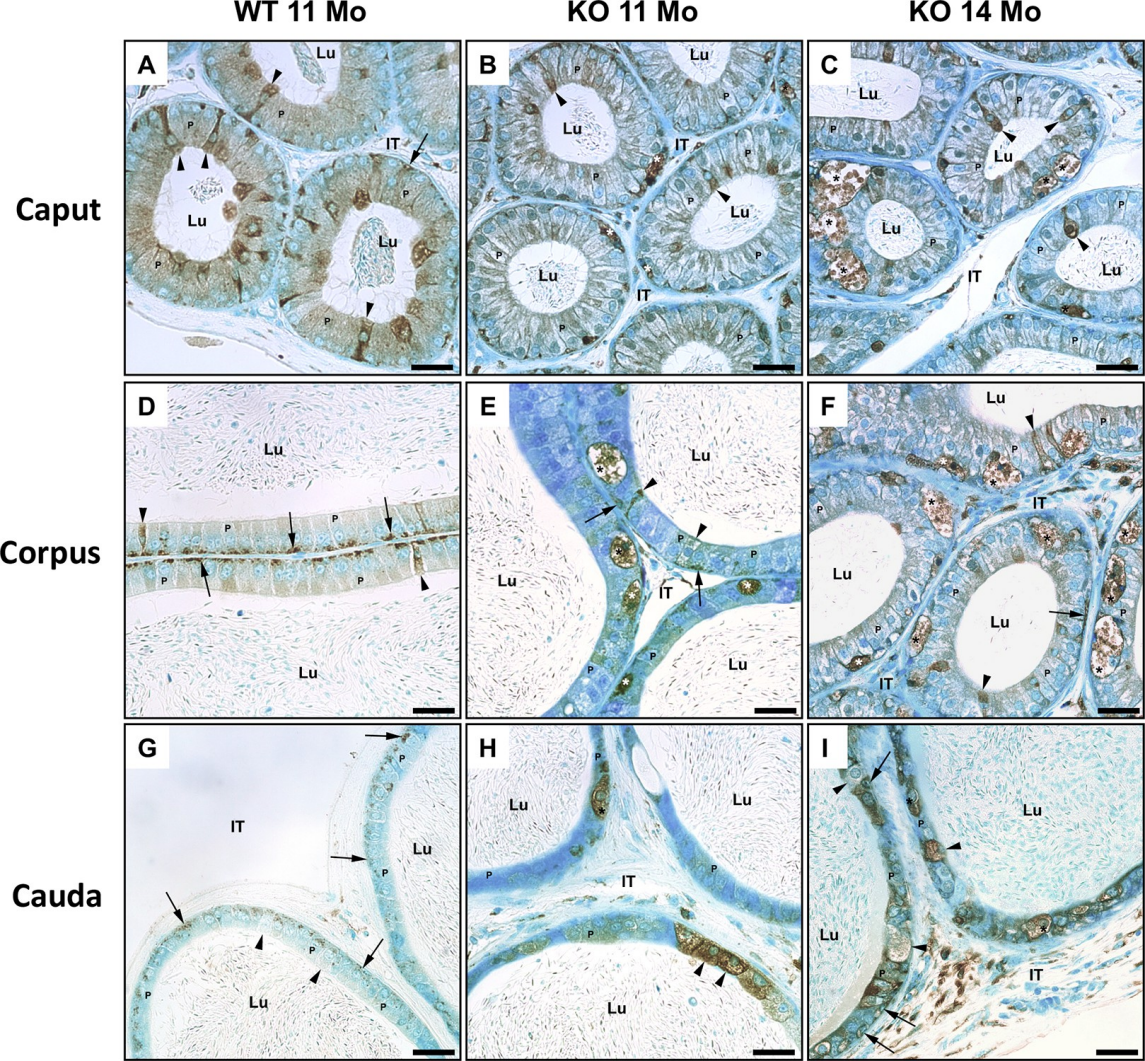
LM immunoreaction of the epididymis of WT (A, D, G) and KO (B, C, E, F, H, I) mice with anti-CathD antibody. Note reactions over principal (P) and basal (arrows). Some clear cells (arrowheads) are reactive. eMPs of KO mice (asterisks) (B, C, E, F, H, I) show intense reactivity. Lu, lumen; IT, intertubular space. Scale bars = 20 μm.

In WT mice, a prominent reaction was observed over clear cells of the caput region (Fig 10A), while the most reactive cell type in the corpus and cauda regions were basal cells (Fig 10D and 10G). The large, dilated eMPs also revealed an intense reaction (Fig 10B, 10C, 10E, 10F, 10H and 10I).

### Abnormalities of epididymal sperm of KO mice by TEM and SEM

**Fig 11:**
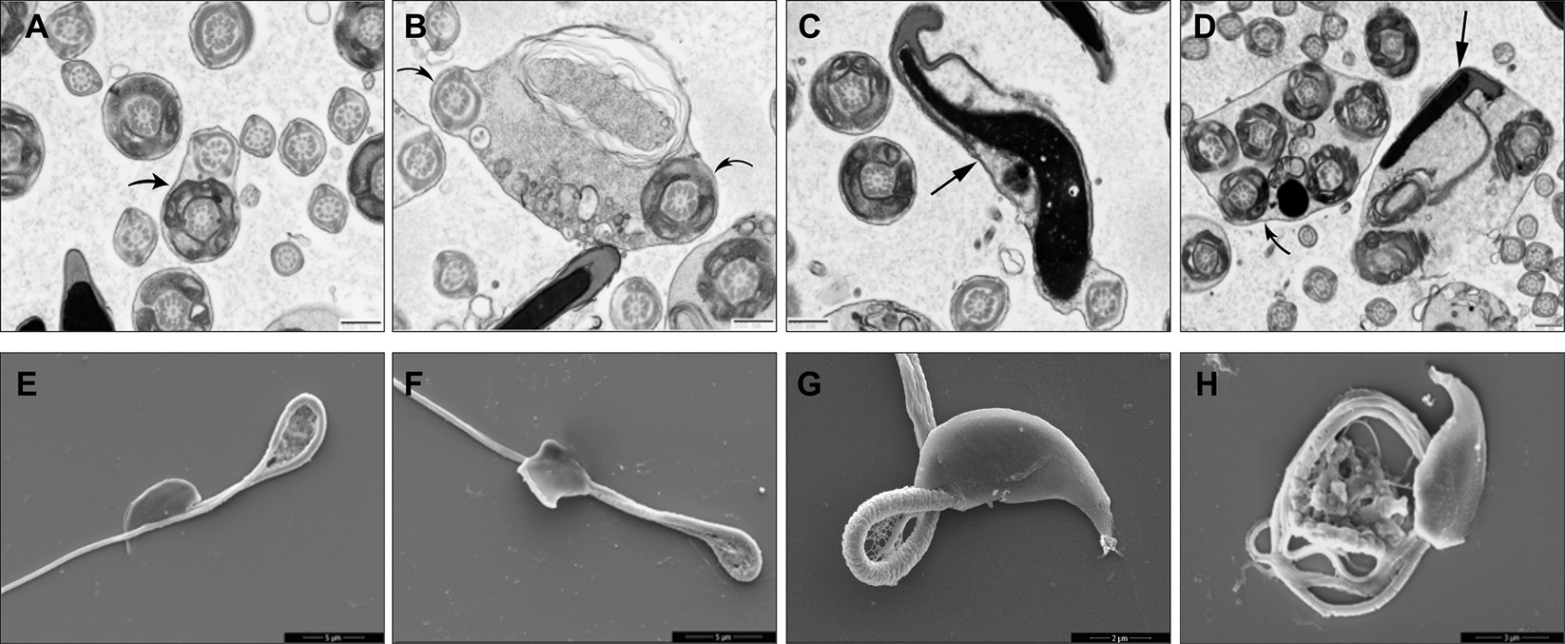
TEM (A-D) and SEM (E-H) of sperm in the epididymal lumen of KO mice. (A-D) Abnormal shapes of sperm heads (large arrows) are noted. Some sperm tails (curved arrows) house more than one cross-sectional profile of the midpiece. Coiling of the sperm tail is noted for some sperm, with few being extreme (E-H). Scale bars A-D = 500 nm; E, F = 5 μm; G = 2 μm; H = 3 μm.

An analysis of the abnormalities of the sperm of KO mice in the epididymal lumen was undertaken by standard transmission (TEM, Fig 11A–11D) and scanning (SEM, Fig 11E–11H) electron microscopy as compared to WT mice and with TEM, cross sections of tails revealed abnormalities in the arrangement and number of outer dense fibers of the tail (Fig 11A, 11B and 11D). Abnormal sperm heads shared the same cytoplasm with sperm tails (Fig 11D). In some cross sections of the tail, the cytoplasm enclosed more than one midpiece (Fig 11A, 11C and 11D), while others presented a midpiece alongside a principal piece (Fig 11A and 11B). SEM analysis confirmed these images in KO mice (Fig 11E–11H). Quantitative analysis of the number of bent tails and several sperm heads revealed statistical differences between WT and KO mice (S7 Fig). * (P< = 0.05) Two-sided t-Test.

### Sperm motility parameters of WT versus KO mice

**Fig 12:**
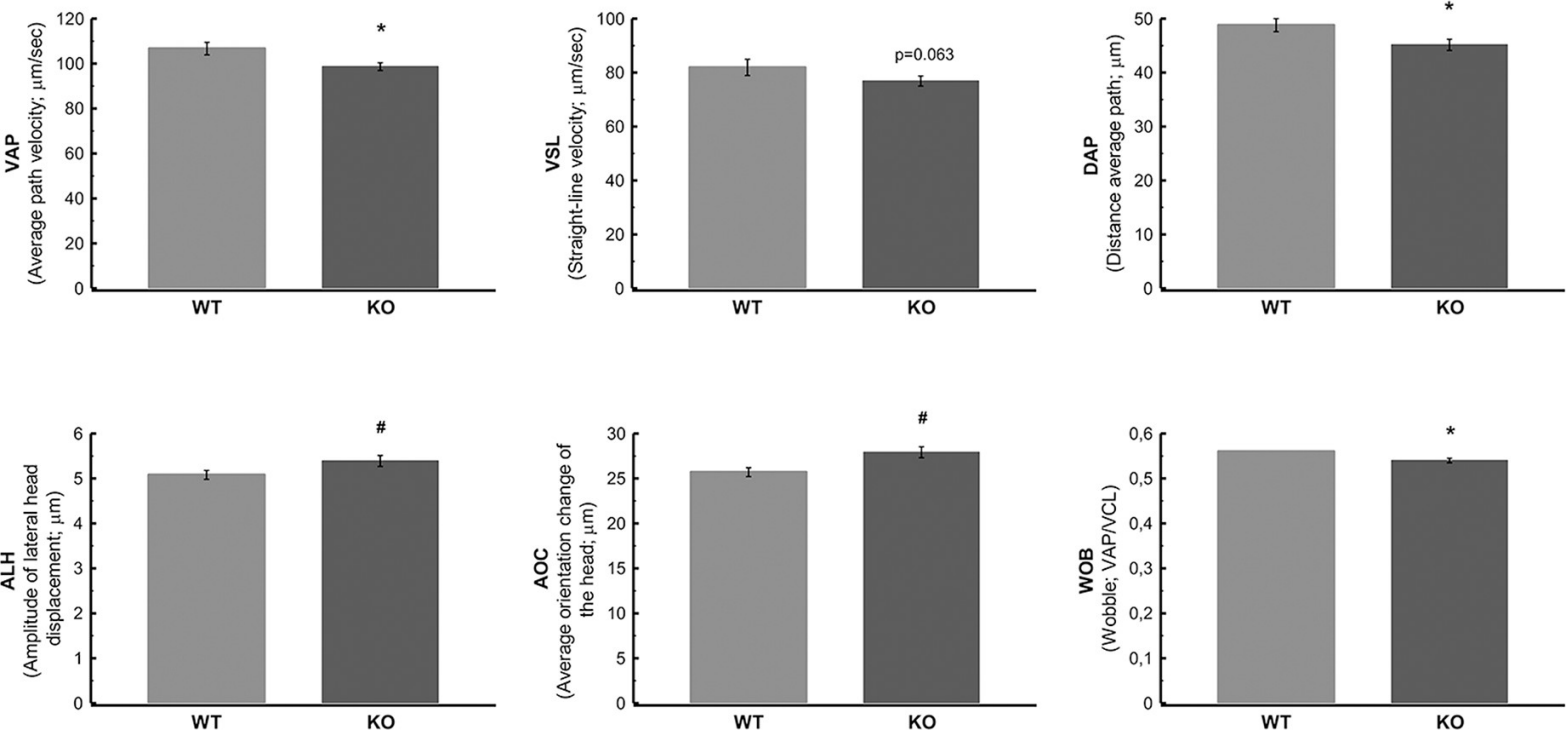
Motility parameters of sperm from cauda epididymis of WT and KO mice were quantified by computer-assisted sperm analysis (CASA). Bars represent the means of VAP (velocity average path, μm/s; P = 0.022); VSL (velocity straight-line, μm/s; P = 0.063); DAP (distance average path, μm; P = 0.032); ALH (amplitude of lateral sperm head displacement, μm; P = 0.046); AOC (average orientation change of the head, μm; P = 0.014) and WOB (wobble coefficient: VAP/VCL; P = 0.014). Error bars indicate the standard error of means. The asterisk (*) indicates a significant decreased pattern change, and # indicates an increased one.

Using the CASA method, sperm motility parameters were analyzed (Fig 12). Although progressive and total sperm motility were not affected in KO mice (S8 Fig), some velocity measures as velocity average path (VAP, μm/s; P = 0.022), velocity straight-line (VSL, μm/s; P = 0.063) and wobble coefficient (WOB: VAP/VCL, where VCL is velocity curved line; P = 0.014), as well as the measure of distance average path (DAP, μm; P = 0.032) were lower compared with WT animals. Also, linearity measures, including the amplitude of lateral sperm head displacement (ALH, μm; P = 0.046) and average orientation change of the head (AOC, μm; P = 0.014), were affected in KO animals. Our results suggest alterations in the velocity, the travelled distance and the movement of sperm in KO mice.

### Effect of *Hgsnat* inactivation on mouse *in vitro* fertilization (IVF)

**Fig 13:**
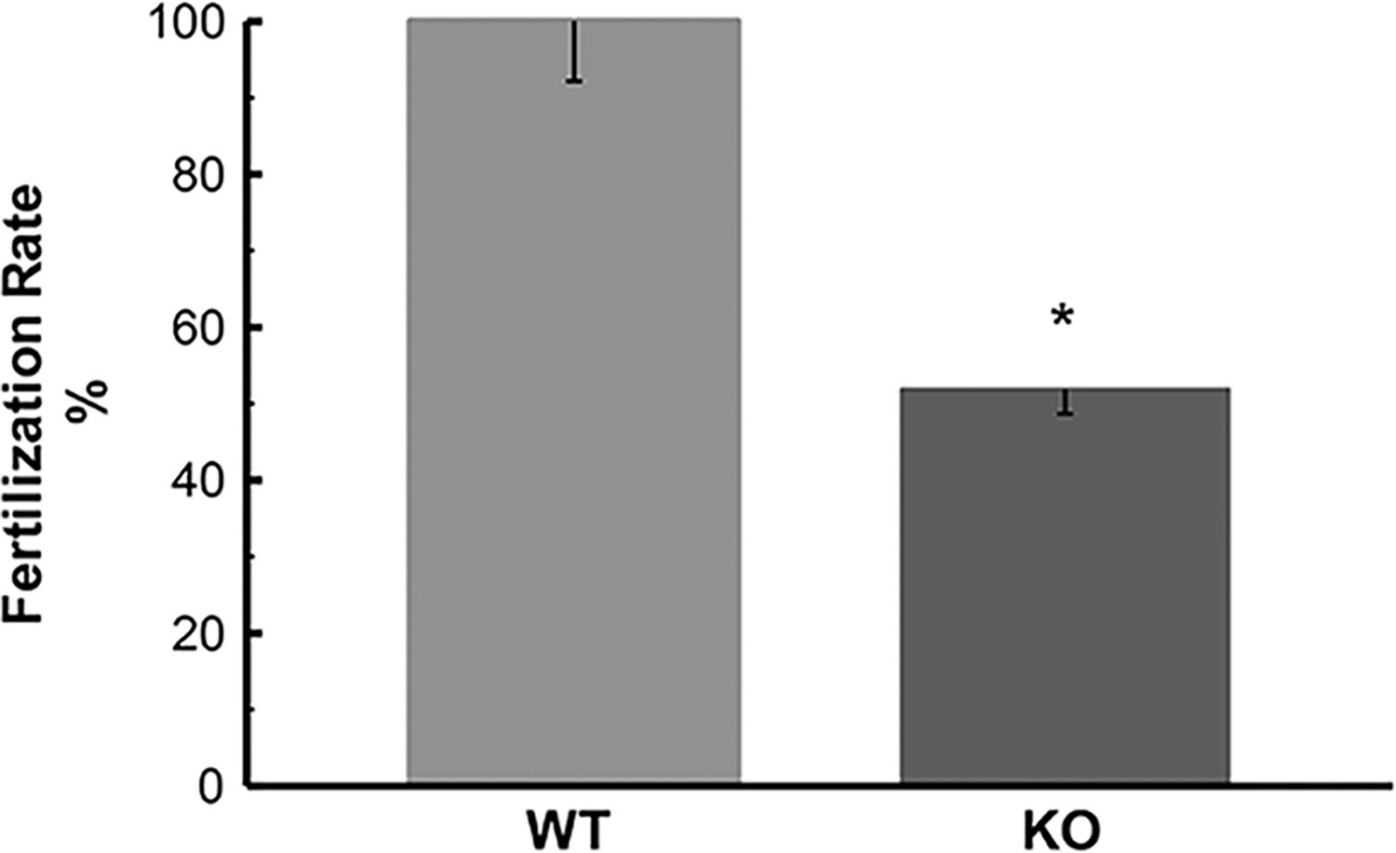
*In vitro* fertilization (IVF) of KO (*Hgsnat−/−*) and WT (*Hgsnat+/+*) mice. IVF rates of WT values were adjusted to 100% for comparative purposes, and experimental values obtained were adjusted accordingly. The block graphs depict the mean percentage of oocytes that were fertilized after incubation with sperm of *Hgsnat* KO animals (n = 33) in IVF medium, as compared to wild type (n = 31). A significant difference at * P = 0,0442 (Fisher exact test, two-tailed, 2x2) was observed between both groups and error bars indicate the standard error of the mean.

Although KO males can produce offspring up to 3 months of age (S9 Fig), we evaluated the fertility rate in these animals at 7 months of age, which is when we evidenced the typical signs of material accumulation in the lysosomes. We found that spermatozoa isolated from KO mouse cauda epididymides have shown a significant decrease in fertilization success rate compared to IVF obtained from WT mice (Fig 13).

## Discussion

In the present study, alterations to the morphology of the testis upon inactivation of the *Hgsnat* gene revealed lysosomes of Sertoli cells to be larger dense structures with a myelinated or granular appearance. Sertoli cells are known to be highly endocytic and phagocytic cells, with the 2 events coordinating with each other to degrade the internalized products [90]. In addition, degeneration of spermatogonia occurs spontaneously during spermatogenesis [54] with apoptotic cells phagocytosed by Sertoli cells [57, 58, 91]. As HS resides on the plasma membrane of multiple types of cells [1, 8, 9, 33], it is suggested that HS is internalized by Sertoli cells during these endocytic/phagocytic events to be degraded, but unable to do so due to the absence of HGSNAT resulting in lysosomal abnormalities.

In affected tubules of the testis, the blood-testis barrier was at times compromised, with adjacent Sertoli cell membranes disrupted or broken at these sites, suggesting impairment of the integrity of the plasma membrane. Damage to the Sertoli cell plasma membrane also resulted in leakage of their organelles and cytoplasmic matrix into the intercellular space of the epithelium. Some tubules lacked germ cells and appeared as Sertoli-Sertoli cell-only animals’ testes. Abnormalities of the integrity of the seminiferous tubule and epithelial profile areas were substantiated by quantitative LM analysis. It has been reported that HSPGs engage with components of the cytoskeleton and focal adhesions (actinin and integrins) [92] and are crucial components not only at the cell surface but with adjacent intracellular regions which influence cell morphology, adhesion, migration and fate decisions [20, 21], a case that could be made for Sertoli and germ cells in the *Hgsnat-/-* model.

However, despite these structural alterations, the phenomenon was not exhibited in the Sertoli cells of all tubules, giving the seminiferous tubules at the LM level an overall on/off appearance. At this point, it is unclear why Sertoli cells of some tubules exhibit serious to moderate abnormalities while others appear normal. However, such a phenomenon has been reported in the testis of other KO mice: huntingtin interacting protein 1 [93] and cystatin-related epididymal spermatogenic protein (CRES, cystatin 8) [82], an avenue for future analysis.

The phenotype observed in MPS IIIC cells can be compared with mucopolysaccharidosis type I (MPS I), a lysosomal storage disease caused by a mutation in the *Idua* gene, which codes α-L-iduronidase (IDUA). In *Idua*-/- mouse model, Nascimento et al. [94] detected abnormalities in Sertoli cells suggestive of incomplete digestion of substrates since vesicles similar to autophagosomes, autolysosomes and lysosomes were detected in a different proportion as seen in wild type mice [95]. In arylsulfatase A KO mice, sulfogalactosylglycerolipid, a major sulfoglycolipid of male germ cells, accumulated in Sertoli cells at 8 months with buildup seen as lysosomal swelling and other cellular abnormalities typical of a lysosomal storage disorder [91].

In the present study, the EDs were structurally altered in their morphology. Both ciliated and nonciliated cells demonstrated a plethora of large pale stained lysosomes supra- and infranuclearly. As both cell types are active endocytic cells [59, 78], the observations suggest an active role in their uptake of HS and eventual degradation, which is compromised by *Hgsnat* gene inactivation.

In the initial segment, *Hgsnat* inactivation leads to a moderate alteration in size and number of the lysosomal population but not to their density. The absence of a significant phenotype implies that HS is not a prominent substrate taken up by the endocytic apparatus of principal cells of this region. The apical and narrow cells of the IS region also did not appear to be affected to any significant degree. Indeed, quantitation of tubule and epithelial profiles of the IS did not reveal any significant differences between WT and KO mice, supporting the lack of a morphological phenotype for principal cells, the major epithelial cell type of the epididymis.

Compared to the few small and irregular lysosomes seen in the supra and infranuclear areas of principal cells of WT mice of the caput, corpus and cauda, those of KO mice were pale stained and grossly altered in size, shape and distribution. Such lysosomes were immunostained with cathepsin D and prosaposin antibodies.

Our present data suggest that as endocytosis continues in principal cells, endosomes form and fuse subsequently with lysosomes to form endolysomal structures. However, as the HS oligosaccharides cannot be degraded due to a lack of HGSNAT, they accumulate in lysosomes. Quantitatively, measurements of the tubule and epithelial profile areas of KO mice demonstrated statistically higher values than WT mice, confirming that major changes were taking place in the epithelium, which could be related to the enlarged lysosomal population of principal cells seen in KO mice.

One of the hallmarks of lysosomal storage diseases is the dramatic increase in the number and size of lysosomes in various cells of different tissues and organs of the body in the absence of other genes [96–101], including that for cells of *Hgsnat* KO mice [1, 33, 36].

*Asm*-/- mice (a mouse model of type A/B Niemann-Pick disease) demonstrate alterations to principal cells of the epididymis with accumulation of lysosomes and changes to their size and shape [49, 102]. In the case of our *Hgsnat* KO mouse, this was reflected mainly in principal cells of the caput, corpus and cauda regions, but not the initial segment. In other lysosomal storage diseases, a region-specific effect was noted for hexosaminidase *Hexa*-/- (Hex A-deficient) mice, whereas in *Hexb*-/- (Hex B-deficient) mice, principal cells of all regions were grossly affected as were clear and narrow cells. Details on adverse effects on the male reproductive system in the case of lysosomal storage diseases can be found in Vuolo et al. [103].

Over the past decade, the epididymal epithelium has been enhanced by the discovery of a previously unrecognized dense network of cells residing within the epididymal epithelium that could be differentiated from COX-1+/cytokeratin 5 (CK5+) basal cells; such cells were classified as epididymal mononuclear phagocytes (eMPs) [71–74, 104, 105]. One of the most profound findings in the epididymal epithelium of the *Hgsnat* KO mouse was a significant alteration to the appearance of these cells.

Aside from their stellate/dendriform morphology, staining properties and antigen-presenting capabilities, a subset of eMPs stained for CD11c (integrin alpha X) and CX3CR1 (a G protein coupled chemokine receptor). Such features classified these epididymal cells as dendritic cells, a cell type known to reside in various lymphoid organs of the body [86]. Additionally, a second cell population was discovered that were F4/80+, which is a marker for M/M cells. The dendritic and M/M cells of the eMPs could be differentiated from each other by specific markers, as well as from basal cells [71–74, 104].

In the present study, we define a population of eMPs that reside at the base of the epithelium distinct from the basal cells as defined by the absence of a reaction with CK5. The eMPs cells reveal contact points with the basement membrane and, in this respect, differ from the non-epithelial halo cells that appear throughout the epithelium, which mainly constitute migrating T lymphocytes and M/M cells [65, 68, 106–109].

Interestingly, at the EM level, the eMPs demonstrated a cytoplasm composed predominantly of a gigantic lysosome consisting of membranous profiles and a finely granular material, leaving little room for the remaining cellular organelles, which appeared stuffed near the nucleus. Curiously, despite the enormous size of the eMPs and their gigantic lysosome, they did not appear to undergo degeneration. As noted for enlarged principal cell lysosomes, thin projections of cytoplasmic matrix poked themselves into the gigantic lysosome but without penetrating their interior.

The presence of such a gigantic lysosome suggested a prominent role for the eMPs in the uptake of HS but in the absence of the HGSNAT enzyme are not able to degrade it, leading to a lysosomal storage effect. The discovery of a functional population of eMPs in the epididymal epithelium that clear defective epithelial cells in the steady-state epididymis, pathogens, and abnormal spermatozoa in the lumen have been raised by several investigators [74, 110–114]. In the dilated intercellular spaces at the base of the epithelium where the eMPs are lodged, membranous profiles and vesicular elements can be found. Hence, the eMPs of KO mice appear to represent activated eMPs that remove such debris from the epithelium, forming a gigantic lysosome that occupies the entire cytoplasm.

One interesting observation is that the eMPs at times were found in close proximity to small spherical halo cells with a small cytoplasmic to nuclear ratio, classified in the literature as T-lymphocytes [66, 68]. Studies on dendritic cells in other tissues have demonstrated a direct interaction with T-lymphocytes [87]. T lymphocytes cross-talk with dendritic cells in multiple tissues and organs [115]. In the epididymis, *in vitro* studies of Da Silva et al. [71] demonstrated that isolated epididymal dendritic cells presented ovalbumin to T cells In our present study, we noted that T lymphocytes *in vivo* reveal a close association with slender stellate cells at the base of the epithelium of WT mice, suggesting that they corresponded to dendritic cells. This affiliation was also noted for the eMPs of KO mice and suggests that a portion of the eMPs represents dendritic cells.

At the LM level, CK5 reactions of WT mice revealed basal cells to be small oval or elongated flattened cells with lateral processes radiating along the basement membrane. At times, basal cells presented thin extensions contacting the lumen, as noted by others [104, 116, 117]. A population of basal cells has been shown to constitute stem cell capability [89, 118–120]. Recent studies demonstrated that purified CD49f+ columnar epithelial cells from day 7 rats could proliferate and differentiate to form epididymal organoids [121]. In *Hgsnat-/-* mice, basal cells appeared larger and more prominent, with some cells reaching the lumen. At the EM level, the prominent basal cells in KO mice possessed numerous pale stained but moderate-sized lysosomes. This enhanced lysosomal profile suggests that HS is also endocytosed by basal cells, which are known to reach the lumen and have endocytic activity [122]. It is possible that internalized HS also result from the removal of basement membrane material and/or debris from the basolateral dilated intercellular spaces.

The role of heparan sulfate proteoglycans (HSPG) as cell-surface receptors of diverse macromolecular cargo has recently been manifested. In many cell types, small membrane vesicles, derived from the either the cell surface and/or multivesicular bodies and referred to as exosomes or extracellular vesicles, deliver a broad spectrum of bioactive molecules to cells, including a variety of proteins, lipids, and nucleic acids such as mRNAs and small non-coding RNA (i.e., miRNAs) [123–125]. Such vesicles can enter cells through HSPG-mediated endocytosis [126]. In this scenario, several possibilities may be put forth as to where the HS originates from that is endocytosed by the epididymal cells of the epididymis.

In the epididymis, exosomes referred to as epididymosomes or apical blebs, have been well-defined as emanating mainly from principal cells and have significant functional roles in sperm maturation [127–131]. Hence exosomes derived from principal cells may be transported to nearby epithelial cells, where they are internalized for specific functions and subsequently degraded by the lysosomal system. Such a paracrine role for epididymal epithelial cells is not unprecedented. Basal cells which reach the lumen interact in a paracrine manner with principal cells to effectively control the proper chloride concentration of the luminal fluid for coordinating events for sperm maturation [132–138]. Also, basal cells, by their apical cell processes, scan and sense the luminal environment of the epididymal epithelium and modulate precise cell functions suggesting a mechanism involving crosstalk between these 2 distinct cell types [72, 104, 139]. Recently clear cells have been implicated in production of exosomes [114], which could interact with principal and basal cells.

Aside from exosomes, the shedding of HSPGs from the cell surface triggered by phospholipases, lipases or a variety of matrix proteases, collectively known as sheddases, represents another level of HSPG-mediated endocytosis [140, 141]. Shed HSPG may act as an extracellular chaperone that transfers ligands in a paracrine manner to cell surface HSPGs on neighboring cells for internalization [142]. Hence, HSPG derived from exosomes and/or sheddases may act in a paracrine manner between principal cells of one region or different regions as well as with basal and clear cells, and in this way, provide HSPG to be internalized and eventually degraded.

The possibility also exists that the sperm may be a source of HSPG. Indeed, heparan sulfate is expressed in spermatozoa and plays an essential role in capturing HIV-1. The spermatozoa-attached virus is efficiently transmitted to dendritic cells, macrophages, and T lymphocytes [143]. Membrane-associated proteins of sperm from rams include alpha-2-heparan sulfate-glycoprotein [144]. Fish sperm head plasma membranes have been demonstrated to contain syndecan (transmembrane heparan sulfate proteoglycan), which may play a role in fertilization [145]. Thus, HS may be on the sperm plasma membrane and released during transit along the epididymis, where sperm protein loss is substantially more predominant than sperm protein gain [146, 147].

In the present study, EM abnormalities were noted for the heads and tails of some sperm in the lumen of the epididymis of *Hgsnat* KO mice. During sperm production in the testis, seminiferous tubules revealed that some Sertoli cells lacked cell processes that hold germ cells in place via ectoplasmic specializations that shape the head of the sperm in a species-related manner. The abnormal head shapes of some sperm suggest that the absence of these processes prevented normal configuration for their heads. In KO mice, abnormal tail configurations were noted by EM for sperm in the epididymal lumen suggesting alterations to their development during spermiogenesis, possibly related to Sertoli cell impairments. Sperm motility parameters were also modified in KO mice. Compared to WT sperm, the KO sperm not only had some velocity and distance parameters decreased, but they also presented problems with parameters that measure the orientation change of the head (AOC) and the head displacement (ALH), which were increased in KO mice; the differences were slight, but still significant. Alterations to the sperm structure, along with adverse effects on sperm motility, could in part, be responsible for the considerable decrease in fertilized oocytes noted *in vitro* for sperm from KO mice. ALH is associated with hyperactivation, and the possibility of hyperactivated spermatozoa would suggest premature capacitation, which is related to low fertility. Indeed, a common thread to many other lysosomal diseases are sperm abnormalities which include, sperm structure, numbers, vitality and motility [91, 94, 102, 148, 149].

### Conclusion

This investigation demonstrates that the catabolism of heparan sulfate in the testis and epididymis is important for the production, quality, and maturation of spermatozoa.

## Supporting information

S1 Data Data sheet and statistical analysis used to build graphs. (XLSX)

S1 Fig EM of seminiferous epithelium (SE) of KO (A-D) mice at 11 or 14 months. In (A, D), Sertoli cells (S) reveal homogeneous electron-dense lysosomes of small and moderate size (arrowheads), along with myelinated and granulated basally located lysosomes (Ly) alongside lipid droplets (Li). In (B), areas of the cytoplasm (cyt) of spermatocytes appear to be bloated. In (B, C), disruption of the blood-testis barrier (curved arrow) is evident, with leakage of organelles and membranous profiles into a resulting expanded intercellular space (ICS). In (B), no Sertoli cell processes are evident between spermatocytes (Spc) and early spermatids (ES); Leydig cells (Ley) appear normal. Sg, spermatogonia; BM, basement membrane; N, Sertoli cell nucleus. Scale bars = 2 μm.
(TIF)

S2 Fig Abnormalities in seminiferous tubules of KO mice. In A, bars represent the percentage of abnormal seminiferous tubules in the testis of WT and KO mice. In B and C, bars represent the profile means of tubule, luminal and epithelial profile areas (μm^2^) of seminiferous tubules stages VI-VIII (B) and stages X-XII (C). Error bars indicate the standard error of means. In A, *P = 0.0374. In B and C, * P values were < = 0.05.
(TIF)

S3 Fig LM of AB-PAS-stained sections of the efferent ducts and IS (A, B), corpus (C-E) and cauda (F) regions of WT (A, C) and KO (B, D-F) mice. In KO mice, the diameter of the efferent duct tubules (ED) is enlarged in size (B) as compared to WT (A), and this is also the case for tubules of the corpus (compare C and D). Large eMPs (asterisks) are evident at the base of the epithelium of the corpus region of KO mice (D, E). Large clear cells (arrowheads) are prominent in the cauda region (F). KO mice show a lumen (Lu) with an abundance of sperm. IT, intertubular space; P, principal cells. Scale bars = 50 μm.
(TIF)

S4 Fig Profile areas of the initial segment (A), caput (B), proximal corpus (C) and middle corpus (D) epididymal tubules. Bars represent the means of tubule, luminal and epithelial profile areas (μm^2^) of each epididymal mentioned region. Error bars indicate the standard error of means. * P values of < = 0.05. ** P values of < = 0.01. *** P values of < = 0.001.
(TIF)

S5 Fig EM of caput (A, B) and corpus (C, D) regions of KO mice. Principal cells (P) are loaded with pale stained lysosomes (Ly) of differing shapes and sizes containing membranous profiles and a finely granular material (A-D), with some lysosomes having dense whorl-like bodies. Lysosomes fill the supra and infranuclear cytoplasm (A-D). Clear cells (CL) show variations in abundance of pale stained lysosomes (A, C, D). Basal cells (BC) contain numerous small pale lysosomes (A, C, D) and elongated lobulated nuclei in (C, D). My, myoid cells; Lu, lumen; N, nucleus; IT, intertubular space; H, halo cell; G, Golgi apparatus. Scale bars = 2 μm.
(TIF)

S6 Fig EM of proximal (A-C) and distal (D) cauda regions of KO mice. Principal cells (P) of the proximal cauda show accumulation of large pale to moderate stained lysosomes supra and infranuclearly (A-C). In contrast, in the distal cauda region (D), they are smaller in size and pale stained. A clear cell (CL) is filled with pale lysosomes (B). Basal cells (BC); My, myoid cells; Lu, lumen; N, nucleus; IT, intertubular space. Scale bars = 2 μm.
(TIF)

S7 Fig Abnormalities in sperm of KO mice. In A, bars represent the mean number of abnormal sperm tails (bent tails) per cauda epididymal area (unit: 245.5 μm^2^). In B, bars represent the mean number of total sperm heads per cauda epididymal area (unit: 245.5 μm^2^). Error bars indicate the standard error of means. Differences among samples were considered significant with a p-value of less than 0.05 (*).
(TIF)

S8 Fig Progressive and total motility of sperm from cauda epididymis of WT and KO mice by computer-assisted sperm analysis (CASA). Bars represent the means of the percentage of progressive and total motility. Error bars indicate the standard error of means. P values less than 0.05 were considered significant.
(TIF)

S9 Fig Litter size of *Hgsnat*-Geo (-/-), *Hgsnat*-Geo (+/-) and Wild Type male and female mice. The number of pups per breeder were graphed. All animals were less than 3 months of age. Error bars indicate the standard error of means. ** P values of < = 0.01. *** P values of < = 0.001. Statistical analyses were performed using Nested One-Way ANOVA-Tukey’s Multiple Comparison Test and GraphPad Prism software (GraphPad Software Inc., USA). P values less than 0.05 were considered significant. *Hgsnat*-Geo (+/-): heterozygous mice.
(TIF)
