# Supplementary figures and images for: Effects of Heparan sulfate acetyl-CoA: Alpha-glucosaminide N-acetyltransferase (HGSNAT) inactivation on the structure and function of epithelial and immune cells of the testis and epididymis and sperm parameters in adult mice
Source: PLoS One. 2023 Sep 27;18(9):e0292157. doi: 10.1371/journal.pone.0292157 (PMC10529547; doi:10.1371/journal.pone.0292157)

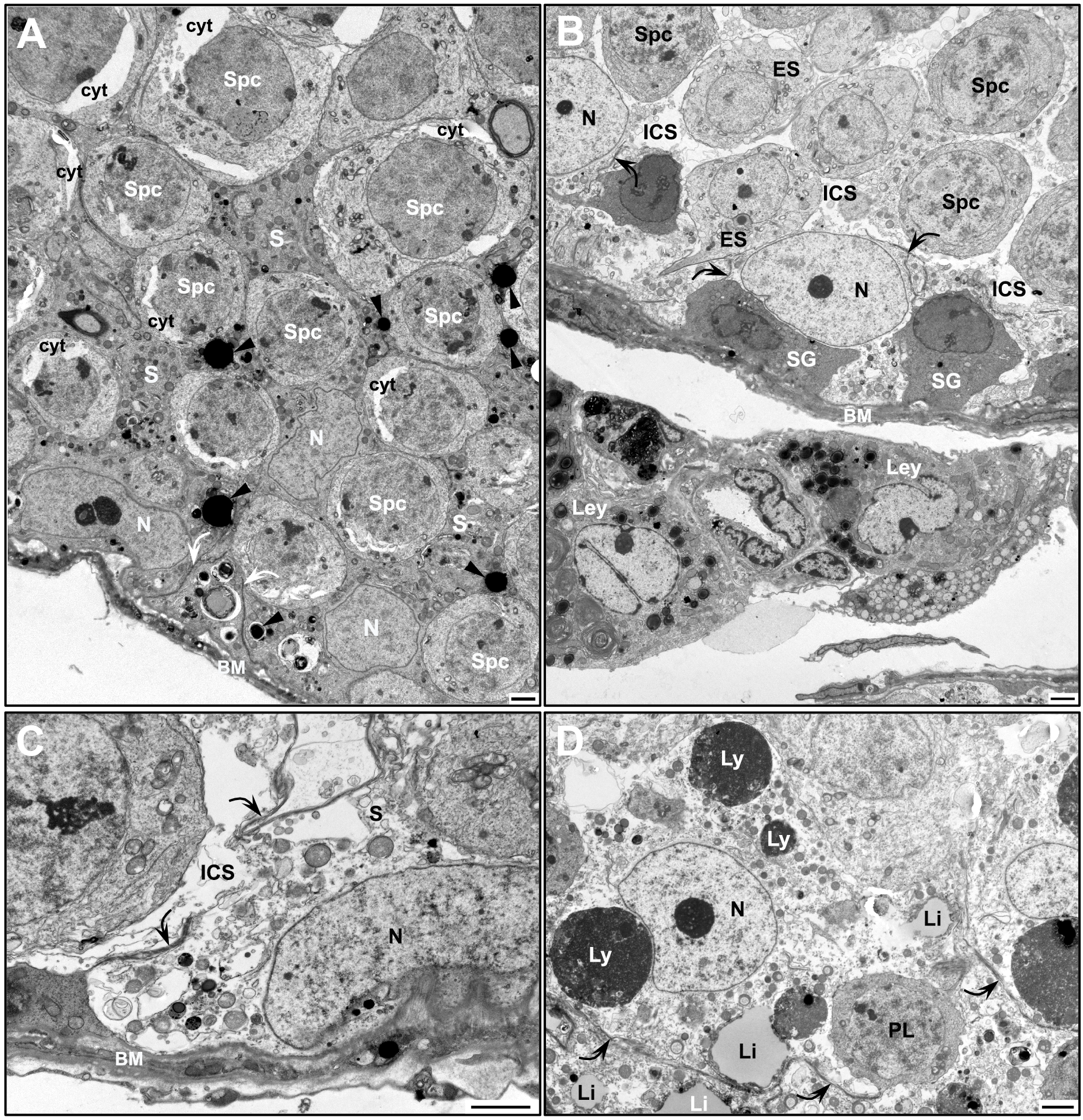

Supplement: S1 Fig — EM of seminiferous epithelium (SE) of KO (A-D) mice at 11 or 14 months. In (A, D), Sertoli cells (S) reveal homogeneous electron-dense lysosomes of small and moderate size (arrowheads), along with myelinated and granulated basally located lysosomes (Ly) alongside lipid droplets (Li). In (B), areas of the cytoplasm (cyt) of spermatocytes appear to be bloated. In (B, C), disruption of the blood-testis barrier (curved arrow) is evident, with leakage of organelles and membranous profiles into a resulting expanded intercellular space (ICS). In (B), no Sertoli cell processes are evident between spermatocytes (Spc) and early spermatids (ES); Leydig cells (Ley) appear normal. Sg, spermatogonia; BM, basement membrane; N, Sertoli cell nucleus. Scale bars = 2 μm. (TIF) [file pone.0292157.s002.tif]

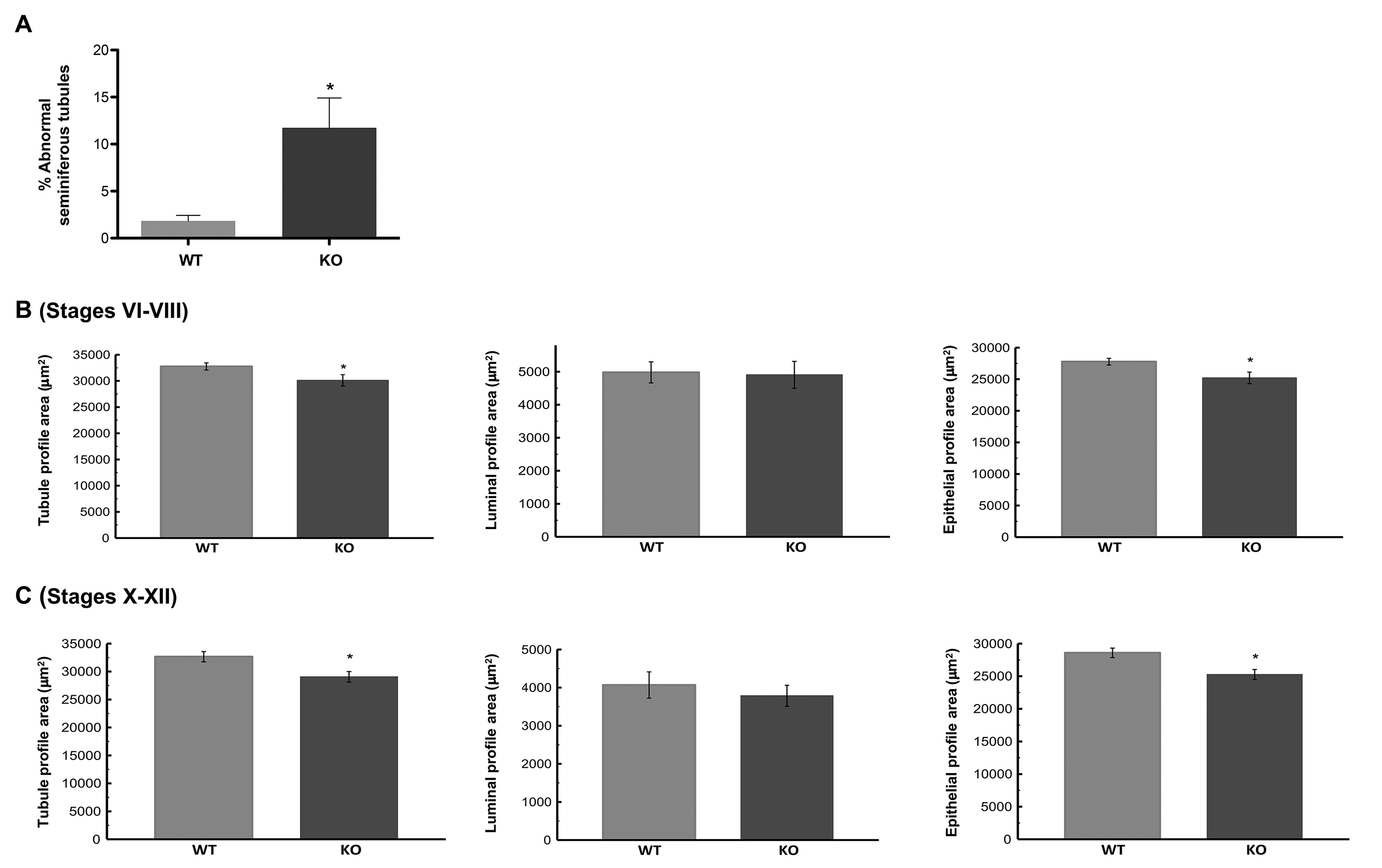

Supplement: S2 Fig — In A, bars represent the percentage of abnormal seminiferous tubules in the testis of WT and KO mice. In B and C, bars represent the profile means of tubule, luminal and epithelial profile areas (μm2) of seminiferous tubules stages VI-VIII (B) and stages X-XII (C). Error bars indicate the standard error of means. In A, *P = 0.0374. In B and C, * P values were < = 0.05. (TIF) [file pone.0292157.s003.tif]

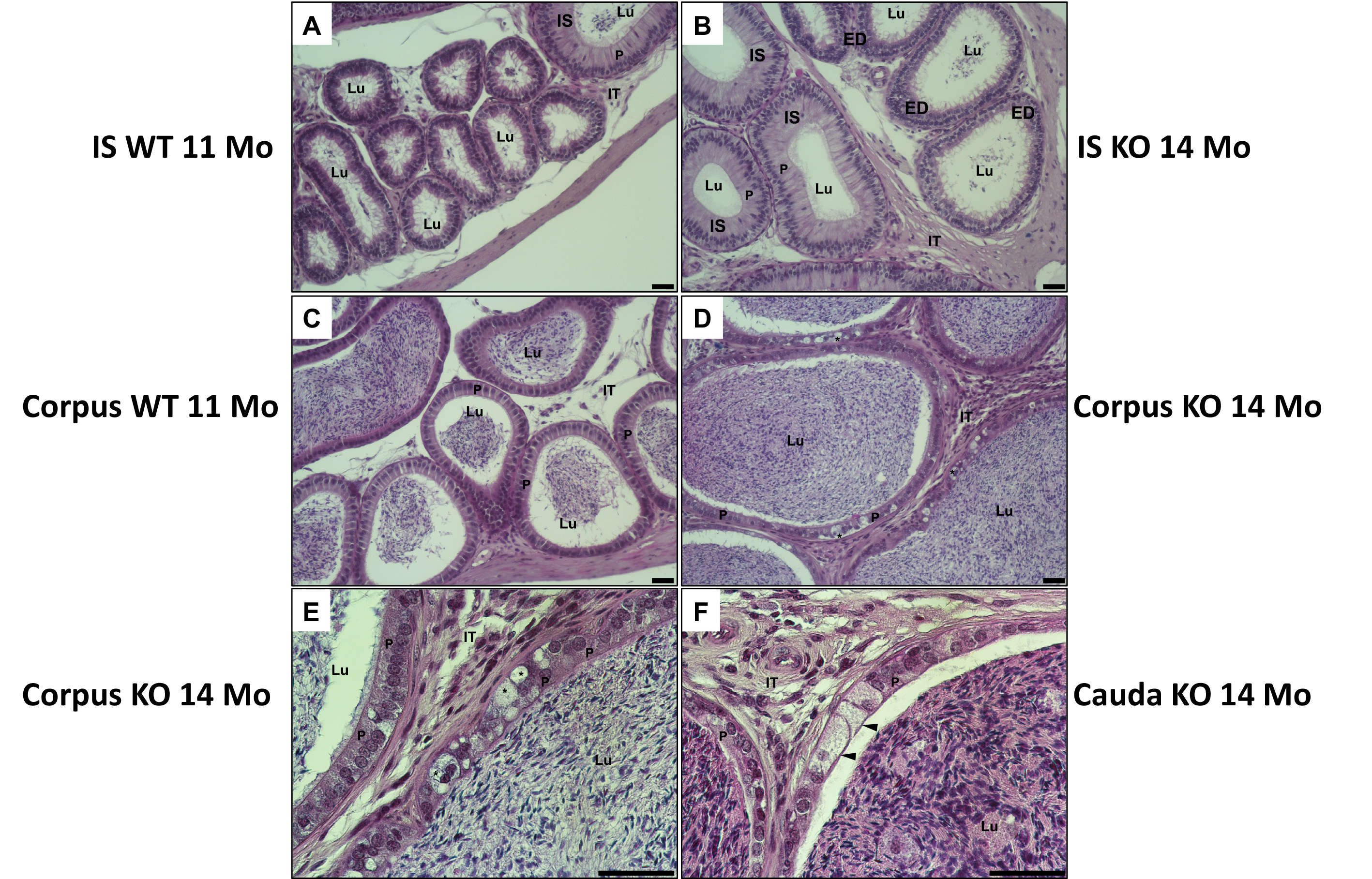

Supplement: S3 Fig — LM of AB-PAS-stained sections of the efferent ducts and IS (A, B), corpus (C-E) and cauda (F) regions of WT (A, C) and KO (B, D-F) mice. In KO mice, the diameter of the efferent duct tubules (ED) is enlarged in size (B) as compared to WT (A), and this is also the case for tubules of the corpus (compare C and D). Large eMPs (asterisks) are evident at the base of the epithelium of the corpus region of KO mice (D, E). Large clear cells (arrowheads) are prominent in the cauda region (F). KO mice show a lumen (Lu) with an abundance of sperm. IT, intertubular space; P, principal cells. Scale bars = 50 μm. (TIF) [file pone.0292157.s004.tif]

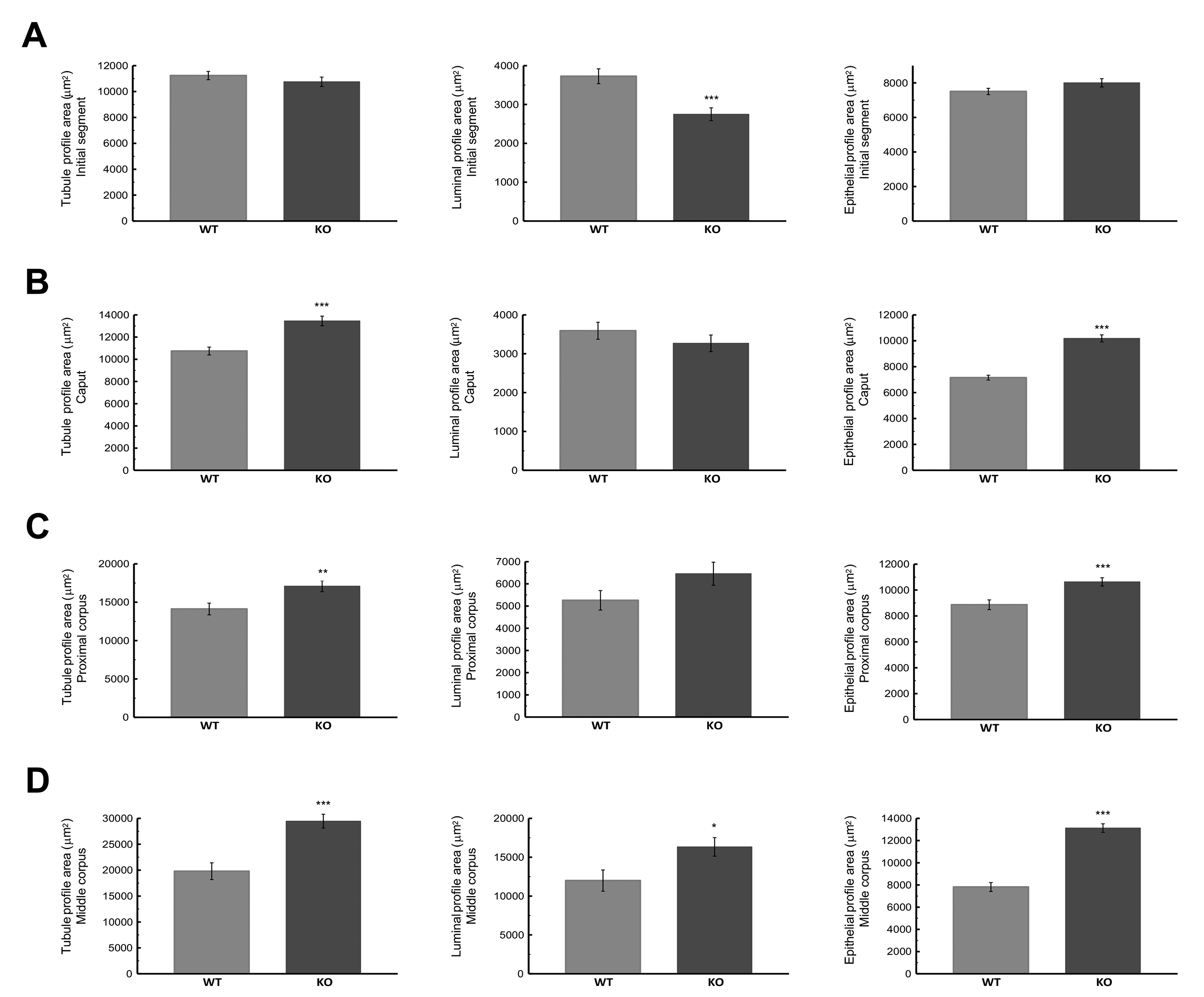

Supplement: S4 Fig — Profile areas of the initial segment (A), caput (B), proximal corpus (C) and middle corpus (D) epididymal tubules. Bars represent the means of tubule, luminal and epithelial profile areas (μm2) of each epididymal mentioned region. Error bars indicate the standard error of means. * P values of < = 0.05. ** P values of < = 0.01. *** P values of < = 0.001. (TIF) [file pone.0292157.s005.tif]

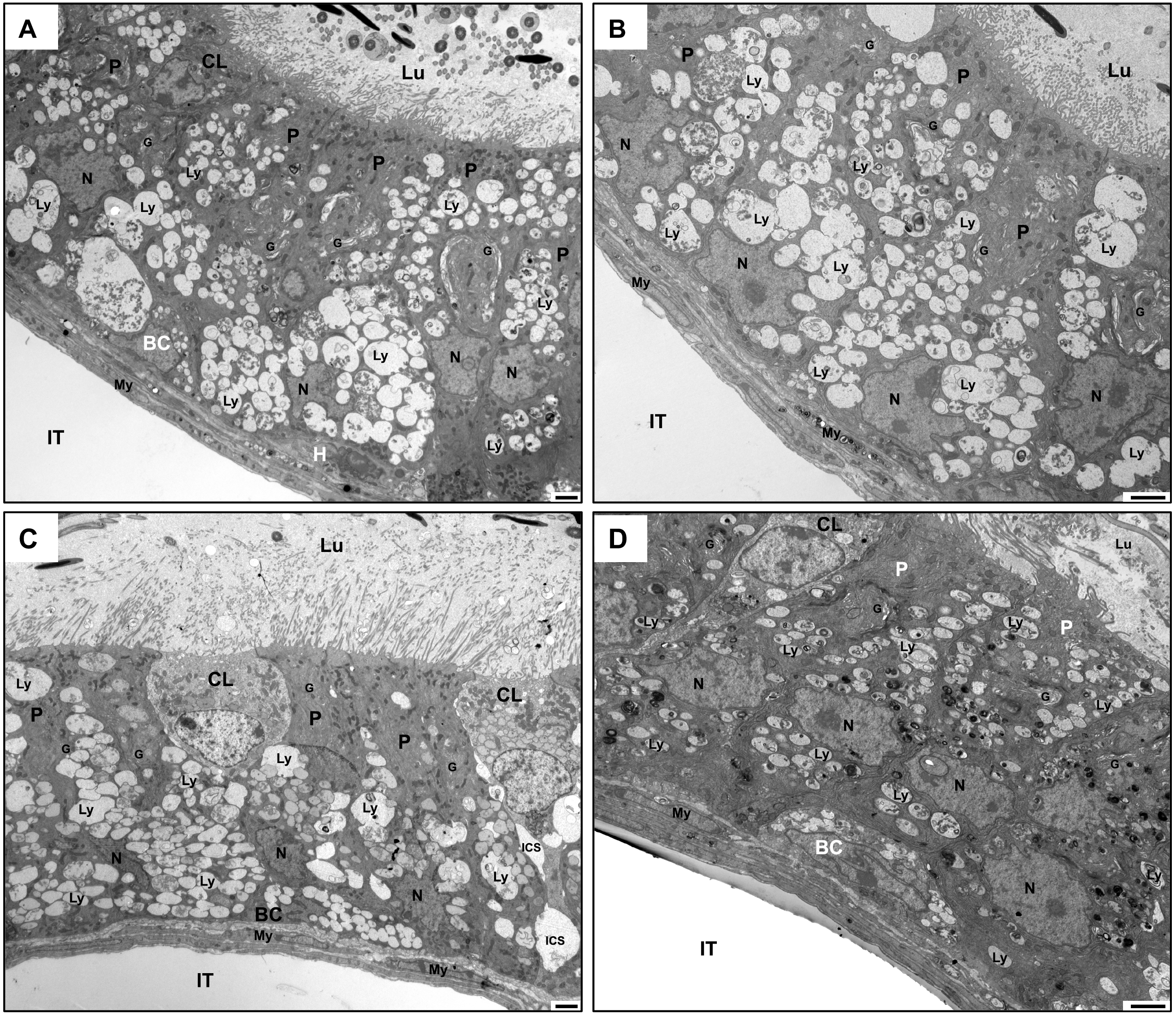

Supplement: S5 Fig — EM of caput (A, B) and corpus (C, D) regions of KO mice. Principal cells (P) are loaded with pale stained lysosomes (Ly) of differing shapes and sizes containing membranous profiles and a finely granular material (A-D), with some lysosomes having dense whorl-like bodies. Lysosomes fill the supra and infranuclear cytoplasm (A-D). Clear cells (CL) show variations in abundance of pale stained lysosomes (A, C, D). Basal cells (BC) contain numerous small pale lysosomes (A, C, D) and elongated lobulated nuclei in (C, D). My, myoid cells; Lu, lumen; N, nucleus; IT, intertubular space; H, halo cell; G, Golgi apparatus. Scale bars = 2 μm. (TIF) [file pone.0292157.s006.tif]

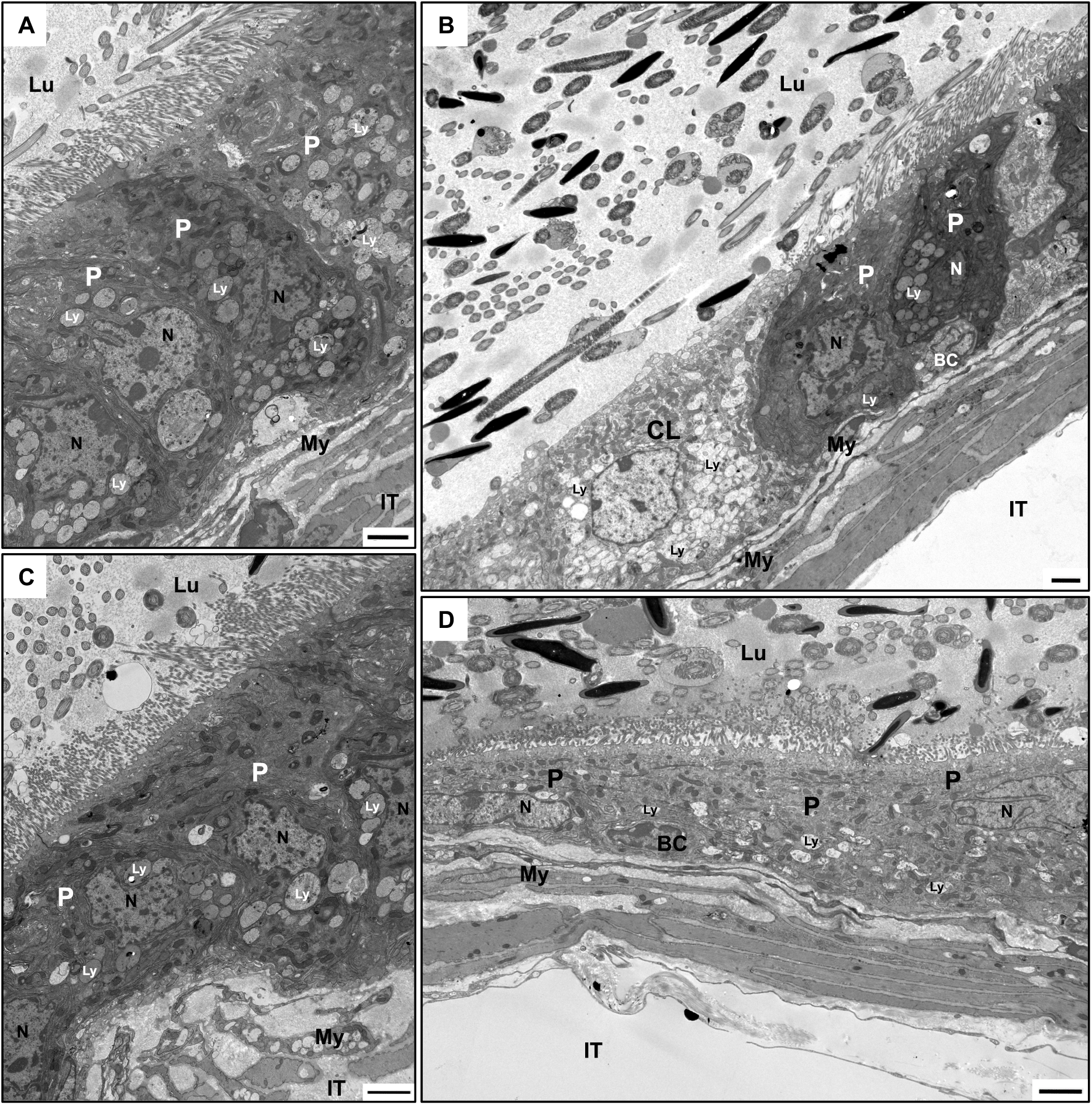

Supplement: S6 Fig — EM of proximal (A-C) and distal (D) cauda regions of KO mice. Principal cells (P) of the proximal cauda show accumulation of large pale to moderate stained lysosomes supra and infranuclearly (A-C). In contrast, in the distal cauda region (D), they are smaller in size and pale stained. A clear cell (CL) is filled with pale lysosomes (B). Basal cells (BC); My, myoid cells; Lu, lumen; N, nucleus; IT, intertubular space. Scale bars = 2 μm. (TIF) [file pone.0292157.s007.tif]

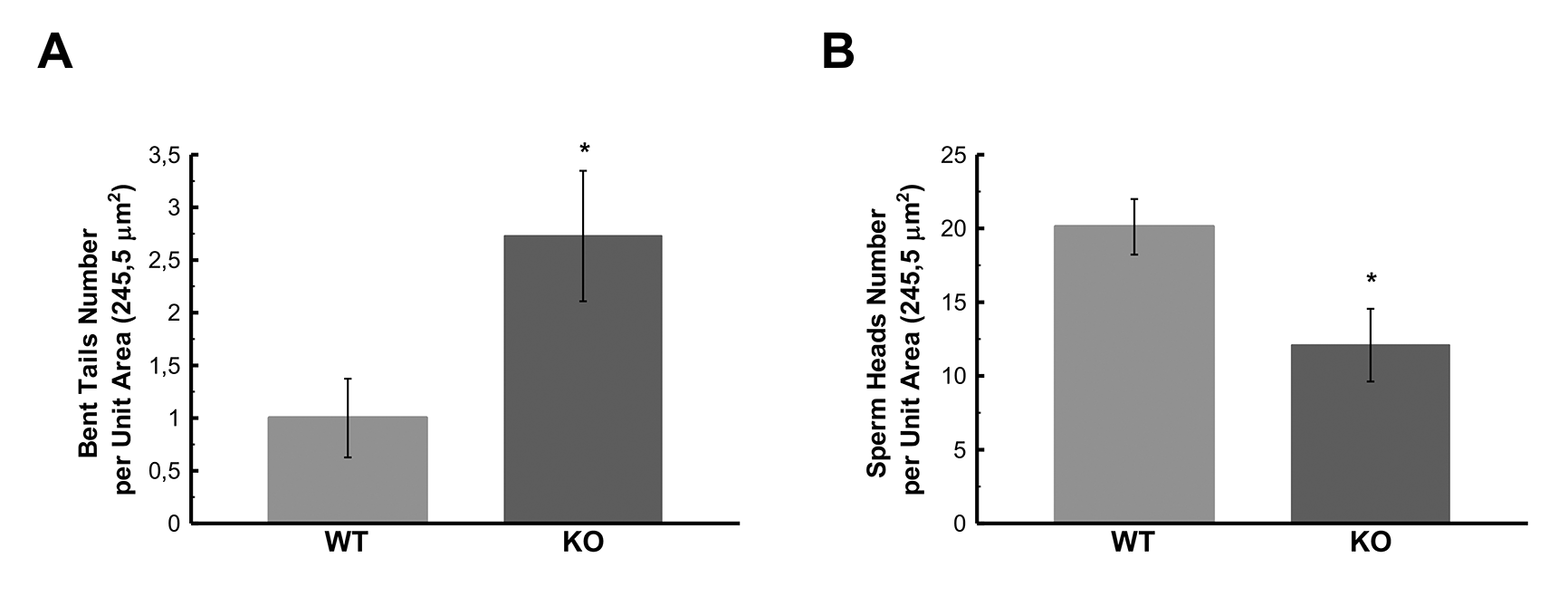

Supplement: S7 Fig — In A, bars represent the mean number of abnormal sperm tails (bent tails) per cauda epididymal area (unit: 245.5 μm2). In B, bars represent the mean number of total sperm heads per cauda epididymal area (unit: 245.5 μm2). Error bars indicate the standard error of means. Differences among samples were considered significant with a p-value of less than 0.05 (*). (TIF) [file pone.0292157.s008.tif]

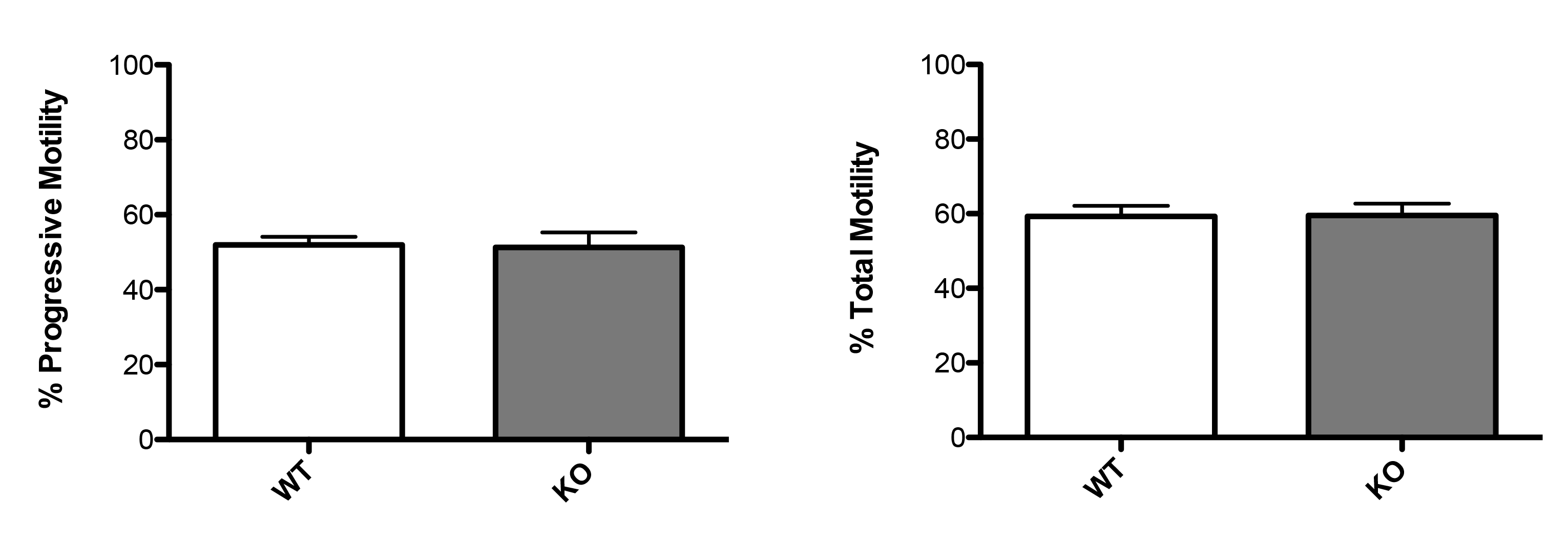

Supplement: S8 Fig — Bars represent the means of the percentage of progressive and total motility. Error bars indicate the standard error of means. P values less than 0.05 were considered significant. (TIF) [file pone.0292157.s009.tif]

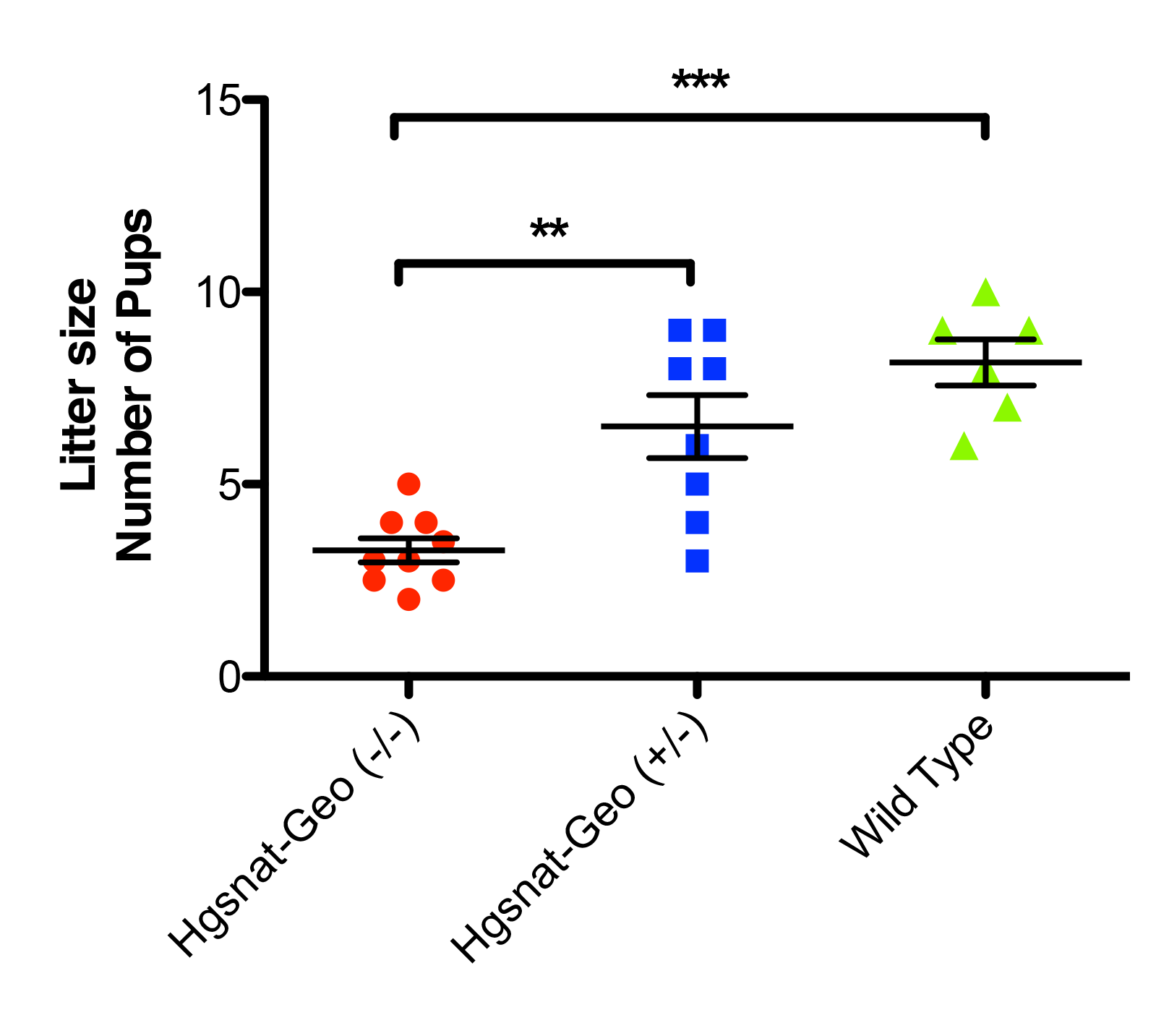

Supplement: S9 Fig — The number of pups per breeder were graphed. All animals were less than 3 months of age. Error bars indicate the standard error of means. ** P values of < = 0.01. *** P values of < = 0.001. Statistical analyses were performed using Nested One-Way ANOVA-Tukey’s Multiple Comparison Test and GraphPad Prism software (GraphPad Software Inc., USA). P values less than 0.05 were considered significant. Hgsnat-Geo (+/-): heterozygous mice. (TIF) [file pone.0292157.s010.tif]
